# Kidney Decellularized Extracellular Matrix Enhanced the Vascularization and Maturation of Human Kidney Organoids

**DOI:** 10.1002/advs.202103526

**Published:** 2022-03-24

**Authors:** Jin Won Kim, Sun Ah Nam, Jawoon Yi, Jae Yun Kim, Jong Young Lee, Seo‐Yeon Park, Tugce Sen, Yoo‐mi Choi, Jae Yeon Lee, Hong Lim Kim, Hyung Wook Kim, Jiwhan Park, Dong‐Woo Cho, Yong Kyun Kim

**Affiliations:** ^1^ Cell Death Disease Research Center College of Medicine The Catholic University of Korea Seoul 06591 Korea; ^2^ School of Life Sciences Gwangju Institute of Science and Technology Gwangju 61005 Korea; ^3^ School of Interdisciplinary Bioscience and Bioengineering Pohang University of Science and Technology Pohang 790‐784 Korea; ^4^ Department of Mechanical Engineering Pohang University of Science and Technology (POSTECH) Pohang Kyungbuk 790‐784 Korea; ^5^ Department of Convergence IT Engineering Pohang University of Science and Technology Pohang 790‐784 Korea; ^6^ Department of Companion Animal Health Daegu Haany University Gyeongsan 790‐784 Republic of Korea; ^7^ Integrative Research Support Center College of Medicine The Catholic University of Korea Seoul 06591 Korea; ^8^ Department of Internal Medicine The Catholic University of Korea St. Vincent's Hospital Suwon 16247 Korea

**Keywords:** extracellular matrix, kidney, organoid, vascularization

## Abstract

Kidney organoids derived from human pluripotent stem cells (hPSCs) have extensive potential for disease modelling and regenerative medicine. However, the limited vascularization and immaturity of kidney organoids have been still remained to overcome. Extracellular matrix (ECM) can provide mechanical support and a biochemical microenvironment for cell growth and differentiation. Here in vitro methods using a kidney decellularized extracellular matrix (dECM) hydrogel to culture hPSC‐derived kidney organoids, which have extensive vascular network and their own endothelial cells, are reported. Single‐cell transcriptomics reveal that the vascularized kidney organoids cultured using the kidney dECM have more mature patterns of glomerular development and higher similarity to human kidney than those cultured without the kidney dECM. Differentiation of *α*‐galactosidase A (GLA)‐knock‐out hPSCs generated using CRISPR/Cas9 into kidney organoids by the culture method using kidney dECM efficiently recapitulate Fabry nephropathy with vasculopathy. Transplantation of kidney organoids with kidney dECM into kidney of mouse accelerates the recruitment of endothelial cells from the host mouse kidney and maintains vascular integrity with the more organized slit diaphragm‐like structures than those without kidney dECM. The kidney dECM methodology for inducing extensive vascularization and maturation of kidney organoids can be applied to studies for kidney development, disease modeling, and regenerative medicine.

## Graphical abstract

The limited vascularization and immaturity of kidney organoids are still to be overcome. Here in vitro methods using a kidney dECM hydrogel to culture hPSC‐derived kidney organoids, which have extensive vascular network with their own endothelial cells and matured nephron‐like structures, are reported. This methodology can be applied to studies for kidney development, disease modeling, and regenerative medicine.

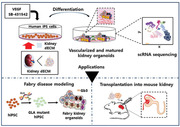

## 1 Introduction

Recent advances in stem cell biology have established several different protocols for generating kidney organoids from human pluripotent stem cells (hPSCs). The hPSC‐derived kidney organoids contain segmented structures with podocytes, proximal tubules, and distal tubules in nephron‐like arrangements.^[^ ^1^, ^2^, ^3^, ^4^ ^]^ Our previous comparative analysis of hPSC‐kidney organoids in vitro and kidney tissue in vivo revealed that kidney organoids recapitulate the development of human kidneys.^[^ ^5^ ^]^ As a consequence, hPSC‐kidney organoids are a promising cell source for kidney tissue regeneration and repair and could be applied as a therapeutic tool in various kidney disease models. Despite biotechnological advances in culturing them, the clinical application of kidney organoids derived from hPSCs has some obstacles. For example, the safety, immaturity, and limited vascularization of kidney organoids are important concerns.^[^ ^6^ ^]^ No existing protocol can generate kidney organoids that completely recapitulate the complex structure and function of kidneys, which limits their efficacy in both kidney disease modeling and regenerative medicine.^[^ ^7^ ^]^

Extracellular matrix (ECM) is the noncellular, extracellular macromolecule component present within all tissues and organs, and it takes the form of a three‐dimensional network.^[^ ^8^ ^]^ ECM molecules and ECM‐related proteins provide a physical substratum for the spatial organization of cells, but they also regulate cell growth and proliferation by interacting with growth factors.^[^ ^9^ ^]^ In kidney development, kidney ECM and ECM‐related molecules regulate ureteric bud (UB) branching morphogenesis, mesenchymal condensation, nephron formation, terminal differentiation of renal tubules, and glomerular basement membrane assembly.^[^ ^9^ ^]^

A hydrogel derived from decellularized tissue‐specific ECM can provide functions similar to those of naturally occurring ECM.^[^ ^10^, ^11^, ^12^ ^]^ Decellularized ECM (dECM)‐based hydrogels could thus provide structural integrity and biochemical cues during tissue engineering.^[^ ^13^, ^14^ ^]^ Kidney dECM hydrogels contain ECM proteins such as collagen IV, laminin, and heparan sulfate proteoglycan and their isoforms to provide a microenvironment similar to that of a normal kidney.^[^ ^15^ ^]^ A kidney dECM hydrogel was reported to enhance the maturation of human umbilical vein endothelial cells and supported endothelial cell growth in flow‐directed microphysiological systems.^[^ ^15^ ^]^

Based on the characteristics of kidney dECM, we hypothesized that kidney dECM could play a role in the maturation of hPSC‐derived kidney organoids, which might make it possible to recapitulate human kidney development. To test this hypothesis, we generated 3D culture protocols to differentiate human inducible PSCs (iPSCs) into kidney organoids using a kidney dECM hydrogel. Single cell RNA sequence analysis revealed that the microenvironment provided by the kidney dECM hydrogel promoted the vascularization and maturation of hPSC‐derived kidney organoids. We also demonstrated that kidney dECM hydrogels support the in vivo growth and maturation of kidney organoids. Our study highlights that kidney dECM hydrogels could be used to more accurately culture kidney organoids. Kidney dECM hydrogels could expand the use of kidney organoids to clinical applications in regenerative medicine or 3D bioprinting to build human kidney‐like structures.

## 2 Results

### 2.1 Gelation and Biochemical Characterization of Kidney dECM Hydrogel

**Figure 1:**
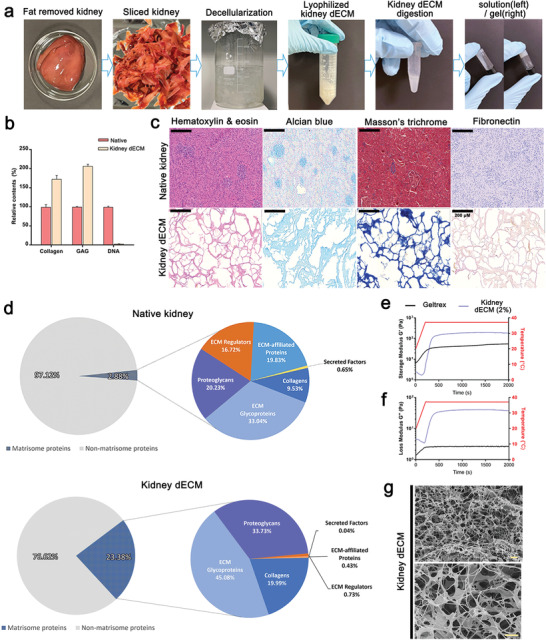
Decellularization process of porcine kidney and characterization. a) The gelation steps of decellularizing porcine kidneys. b) Paired column comparison of collagen, glycosaminoglycan (GAG), and DNA before (Native) and after kidney tissue decellularization (kidney dECM) (*n* = 3). c) Representative images of native kidney and kidney dECM gel stained with hematoxylin & eosin, Alcian blue, Masson's trichrome, and Fibronectin. Scale bar = 200 µm. d) Graph of proteins identified by liquid chromatography–tandem mass spectrometry (LC–MS/MS) in native kidney and kidney dECM. e,f) Storage and loss modulus comparison of kidney dECM pre‐gel and Matrigel. g) Scanning electron microscopy images of the kidney dECM hydrogel at 10 000× (left) and 30 000× magnification (right). Scale bar = 2 µm.

To optimize the process for producing a kidney dECM hydrogel, we designed a 5‐step protocol: i) harvesting porcine kidney tissue; ii) decellularization; iii) lyophilization; iv) digestion; and v) neutralization (**Figure** **1**a). To verify that the kidney tissue was effectively decellularized, we used dsDNA quantification, in which remnant DNA content represents remaining cellular components in the tissue. The DNA content in the kidney dECM was measured to be 0.64 ng of DNA/mg, which is equivalent to 2.29% of the DNA in native kidney tissue; thus, most cellular components were successfully removed (Figure 1b). Using three kidney dECM samples obtained from repeated decellularization processes, we quantified collagen and glycosaminoglycans (GAGs) using the hydroxyproline assay and dimethylmethylene blue (DMMB) assay, respectively. Compared with an identical mass of native kidney tissue, the kidney dECM contained around 174.43% and 207.12% of collagen and GAGs, respectively (Figure 1b).

Hematoxylin and eosin (H&E) staining revealed no visible cellular components in the kidney dECM (Figure 1c; Figure S1, Supporting Information). The remaining fibronectin and collagen components were also assessed visually using Alcian blue, anti‐fibronectin, and Masson's trichrome staining (Figure 1c; Figure S1, Supporting Information). Those tests showed that fibronectin and collagen, the main ECM components in the kidney, were well preserved in the decellularized kidney tissue. Taken together, our data indicate that the decellularizing process worked efficiently by removing cellular components and maintaining ECM components, producing a functional material with a native renal environment. The growth factor and cytokine concentrations in kidney dECM were compared with those in native kidney tissue, as shown in Table S1, Supporting Information.

To determine and compare protein composition between kidney dECM and native kidney before decellularization, we performed liquid chromatography–tandem mass spectrometry (LC‐MS/MS) (Figure 1d). LC‐MS/MS proteomic analysis showed that the matrisome protein components increased by almost tenfold (≈23.38%) in kidney dECM, while a vast majority of the proteins in the native kidney was non‐matrisome proteins (≈97.12%) (Figure 1d; Tables S2,S3, Supporting Information). Despite the reduction in growth factor in kidney dECM, the core matrisome protein composition including collagens, proteoglycans, and ECM glycoproteins were dramatically increased in kidney dECM and play a significant role in cell growth and maturation (Figure 1g; Tables S1–S3, Supporting Information). Heparan sulfate proteoglycan (HSPG) 2 constituted up to 30% of matrisome proteins of kidney dECM (Figure 1g; Tables S2,S3, Supporting Information), while Matrigel mostly contain ECM glycoproteins such as laminin, collagen IV, and enactin.^[^ ^16^ ^]^ HSPG has the ability to sequester soluble growth factors important to cell viability and to transduce signals to maintain endothelial cell stasis, a contributing angiogenic factor during differentiation.^[^ ^17^, ^18^ ^]^

### 2.2 Rheological and Physical Characterization of the Kidney dECM Hydrogel

The rheological and mechanical properties of the microenvironment are critical to organoid culture. To assess the rheological characteristics of the kidney dECM hydrogel, 2% kidney dECM pre‐gel was prepared and loaded onto a Discovery Hybrid Rheometer‐2 (TA Instruments, USA), which has a steel 20 mm parallel plate geometry. An oscillatory temperature sweep test was performed to evaluate the gelation kinetics, both storage modulus and loss modulus, of the kidney dECM pre‐gel and Matrigel from 20 to 37 °C at a heating rate of 5 °C min^−1^ (Figure 1e,f). As the sweep temperature approached and stayed at 37 °C, an increase in the storage modulus was observed in both the kidney dECM pre‐gel and the Matrigel, indicating the presence of collagenous fibers along with its ability to thermally cross‐link and to make a transition from pre‐gel to a stabilized hydrogel state. To investigate the surface microstructure of the kidney dECM hydrogel, we used scanning electron microscopy (SEM). Those results show an interwoven network of collagen fiber amalgamates and other ECM components present in native kidney tissue (Figure 1g). These findings indicate that the kidney dECM hydrogel can act as a supporting material providing microenvironment for differentiation of kidney organoids.

### 2.3 Protocols for the Differentiation of Human Kidney Organoids Based on Kidney dECM Hydrogel

**Figure 2:**
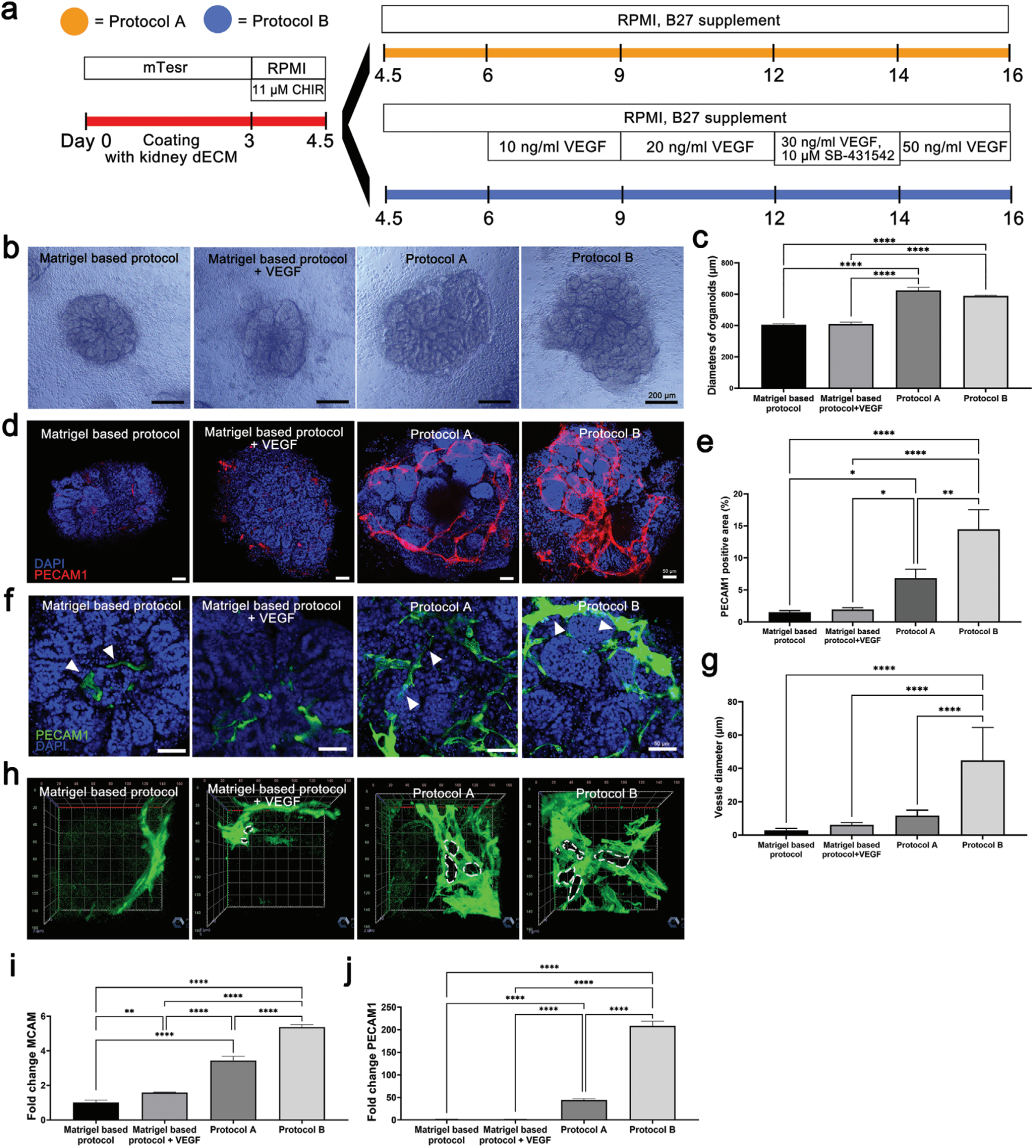
Kidney organoids differentiation using kidney tissue decellularized ECM gel. a) Protocols for kidney organoids differentiation based on kidney dECM hydrogel. b) Representative bright field images of the morphology of kidney organoids cultured by Matrigel‐based protocol, Matrigel‐based protocol+VEGF, protocol A and protocol B. Scale bar = 200 µm. c) Diameters of kidney organoids differentiated by four protocols (*n* = 10). d,e) Representative confocal images of PECAM1 and quantification of the percentage of PECAM1 positive area. Scale bar = 50 µm (*n* = 3). f,g) Representative confocal fluorescence images showing PECAM1 and the quantification of the diameter of the kidney organoids vessels. Scale bar = 25 µm (*n* = 3). h) Representative 3D confocal fluorescence images showing PECAM1. i,j) qRT‐PCR analysis of MCAM and PECAM1 in kidney organoids differentiated by Matrigel‐based protocol, Matrigel‐based protocol + VEGF, protocol A and protocol B kidney organoids (*n* = 3). Values are mean ± SEM. *p < 0.05, **p < 0.01, ***p < 0.001, ****p < 0.0001, measured by one‐way ANOVA with Tukey's multiple comparisons test.

To test the effect of kidney dECM on kidney organoid differentiation, we developed two 3D culture systems to differentiate from human iPSCs into kidney organoids using the kidney dECM hydrogel (**Figure** **2**a). In protocol A, each well of a 24‐well plate was coated with diluted kidney dECM (0.1%) and then dissociated, undifferentiated human iPSCs were placed evenly. After 24 h, we added 1.5% Matrigel, sandwiching the hPSCs between a lower layer of kidney dECM and an upper layer of Matrigel. The sandwiched hPSCs formed compact, ball‐like colonies within 48 h and then developed internal cavities. On day 3, the cells were treated with CHIR, and then they were fed with an appropriate medium every 2–3 days to promote kidney organoid differentiation, as described in Figure 2a.

Protocol B began the same as protocol A, but we added VEGF on days 6, 9, 12, and 14 to enhance the vascular network and SB‐431542 on day 12 to enhance podocyte differentiation. Kidney organoids were efficiently generated by both protocols A and B (Figure 2a).

Next, we examined the characterization of the coated kidney dECM (Figure S2a–d, Supporting Information). To determine how long the coated kidney dECM remained in the well, we coated the well with 0.1% kidney dECM without seeding iPSCs and tested the degradation of the dECM (Figure S2a,b, Supporting Information). Collagen I, one of the major proteins of kidney dECM, remained at 18 days after coating, at the end of differentiation, which suggest that kidney dECM maintains its effect throughout the differentiation period (Figure S2a,b, Supporting Information). We also investigated whether kidney organoids remodeled kidney dECM. Immunofluorescence analysis for deposition of structural basal lamina components fibrillar protein collagen IV showed substantially higher and more widespread expression of COL IV in groups with protocol A or B compared to the Matrigel‐based protocol group (Figure S2c, Supporting Information). qPCR analysis revealed that a set of integrins involved in the binding to collagens (ITGB1) as well as collagen and fibronectin are overexpressed in the groups with protocol A or B (Figure S2d, Supporting Information). These findings suggest that kidney organoids interact with dECM to produce dECM remodeling.

### 2.4 The Kidney dECM Hydrogel Enhanced the Formation of a Vascular Network in Human Kidney Organoids

To demonstrate the effects of the kidney dECM hydrogel in the differentiation of kidney organoids, we generated kidney organoids using protocols A and B and compared their phenotypes with those of kidney organoids generated using Matrigel without kidney dECM (Matrigel‐based protocol group). Kidney dECM contains VEGF (Table S2, Supporting Information), which enhances vascularization of kidney organoids. To clarify the effect of kidney dECM on differentiation of kidney organoids, we added a group with kidney organoids generated using a Matrigel‐based protocol with VEGF (Matrigel‐based protocol + VEGF group) (Figure 2a).

Bright field microscopy revealed that that the kidney organoids generated by protocols A and B had tubular structures, and their size was greater than the kidney organoids without kidney dECM (Figure 2b,c; Figure S5, Supporting Information). Comparing the size of mouse kidney, the kidney organoids generated by protocol A was about 17 times smaller than that of normal mouse kidney (Figure S3, Supporting Information). Then we investigated the effect of kidney dECM on the vascularization in the kidney organoids. The vascularization of PECAM1+ endothelial cells was limited in the Matrigel protocol group, which is slightly increased in Matrigel‐based protocol + VEGF group. The vascularization was extensively increased in the kidney organoids generated by protocol A, and accelerated in those generated by protocol B (Figure 2d; Figure S4a, Supporting Information). The areas and diameters of the PECAM1‐positive vascular structures was enhanced in protocol A and accelerated in protocol B (Figure 2d–h). Kidney organoids differentiated from another human iPSC cell line, CMC 11, showed the same vascularization pattern as the WTC11 human iPSC cell line (Figure S4b, Supporting Information). Real‐time quantitative polymerase chain reaction (RT‐qPCR) showed that protocols A and B upregulated the gene expression of the vascular marker PECAM1 and its precursor, MCAM, compared with their expression in the control kidney organoids (Figure 2i, j).

In Figure S2a,b, Supporting Information, we show that kidney dECM maintained its effect throughout the differentiation period despite the presence of coating. To determine the time of formation of endogenous endothelial‐progenitors and endothelial‐like cells, we stained endothelial cells during differentiation according to protocol. PECAM1+ endothelial cells were observed at 7 days after differentiation in the protocol A or B groups, which is earlier than in the control group (Figure S5, Supporting Information). PECAM1+ endothelial cells subjected to protocol A or B extensively increased at day 12 and continued to do so until the end of differentiation; those in the control group showed limited expression (Figure S5, Supporting Information). Taken together, our data suggest that the persistent effect of kidney dECM through the differentiation period promotes vascularization from a relatively early stage through the late stage of kidney organoid differentiation.

### 2.5 Kidney dECM Hydrogel Enhanced Glomerular Vascularization and Morphogenesis of Kidney Organoids

**Figure 3:**
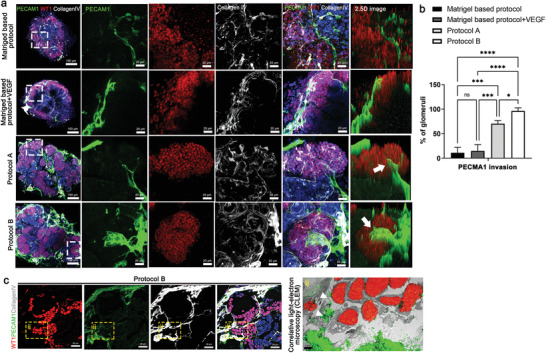
Enhanced vascularization of kidney organoids cultured on kidney dECM by protocol B. a) Representative images of immunofluorescent staining of WT1 (podocyte), PECAM1 (vascular network), and CollagenIV (basement membrane) in kidney organoids. Invasion of PECAM1+ endothelial cells into WT1+ podocytes clusters (white arrow). Scale bar = 100 µm, 20 µm. b) Quantification of the percentage of PECAM1 invasion into the glomerular‐like structure in the kidney organoid (*n* = 3). c) Representative images of a correlative light‐ and electron‐microscope (CLEM) study by overlaying a confocal microscopy image stained to reveal PECAM1, WT1, and collagen IV with an electron microscope (EM) image of the same structures in the kidney organoids (panel ii). Scale bar = 20 µm for immunofluorescent staining and scale bar = 2 µm for EM. Values are mean ± SEM. NS, no significance, *p < 0.05, ***p < 0.001, ****p < 0.0001, measured by one‐way ANOVA with Tukey's multiple comparisons test.

Kidney organoids have previously shown the presence of nascent vascular endothelial cells that surround the glomerular structures, but they lack a proper vascular pattern.^[^ ^6^ ^]^ After transplantation of kidney organoids into an animal, host‐derived vascularization promotes glomerular vascularization.^[^ ^6^ ^]^ To determine whether kidney dECM–induced vascularization of organoids in vitro extended to the glomerular compartments, we used confocal imaging to quantify NPHS1+ or WT1+ podocyte clusters invaded by PECAM1+ vascular structures in kidney organoids. The PECAM1+vascular invasion into the glomerular structure was rarely observed in the Matrigel‐based group or Matrigel + VEGF group, although vascularization was observed surrounding the glomerular structure (**Figure** **3**a). With protocol A, PECAM1+vascular invasion into the glomerular structure was observed in some portion of the glomerular compartment (Figure 3a,b). Protocol B, which adds VEGF and SB‐431542 to protocol A, showed extensively enhanced vascularization wrapping the glomerular structure and larger diameter PECAM1+ vessel‐like structures (Figure 3a,b). Notably, small branches from large PECAM1+ vessel‐like structures invaded NPHS1+ or WT1+ glomerular structures (Figure 3a,b; Figure S6a–c, Supporting Information).

To precisely determine the PECAM1+ vascular invasion into glomerular structure, we performed a correlative light‐ and electron‐microscopy (CLEM) study by overlaying a confocal microscopy image stained with PECAM1, WT1, and collagen IV with an electron microscope (EM) image of the same structures in the kidney organoids (Figure 3c). The overlay of confocal microscopy and EM images confirmed that the PECAM1+endothelial cells infiltrated into the WT1+ podocyte clusters (Figure 3c). Considering that WT1 is expressed not only in podocytes but also in renal progenitor cells during kidney development, these findings can be interpreted as the PECAM1+ vascular invasion into WT1+cell clusters rather than as capillary loop formation in kidney organoids.

We also examined the podocyte apico‐basal polarity with endothelial and basal membrane distribution within the glomerular‐like structures in the protocol B group. Apical aspect podocytes stained with PODXL were present above the collagen IV+, GBM compartment, and interface with PCAM1+ endothelial cells, suggesting proper correlation of podocyte apico‐basal polarity (Figure S6d, Supporting Information).

**Figure 4:**
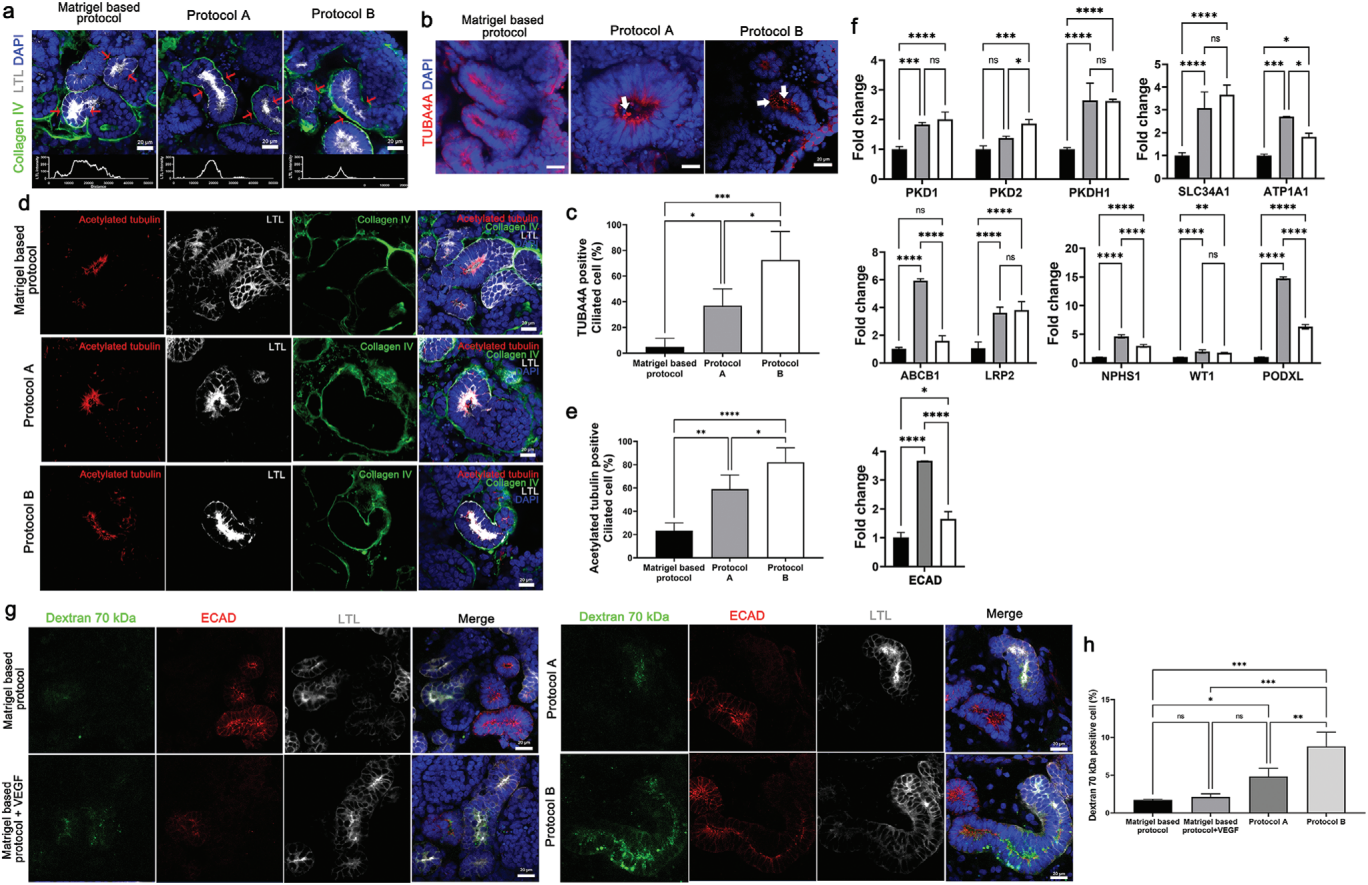
Enhanced the maturation of tubular structure of kidney organoids cultured on kidney dECM. a) Representative images of immunofluorescent staining of LTL and collagen IV in kidney organoids. LTL across the line scan indicated by red arrows above each confocal image. Scale bar = 20 µm (*n* = 2). b) Representative images of immunofluorescent staining of TUBA4A in kidney organoids. White arrows indicated ciliated cells. Scale bar = 20 µm. c) Quantification of the percentage of TUBA4A positive ciliated cell (*n* = 4). d) Representative confocal fluorescence images showing acetylated tubulin, LTL, and collagen IV in kidney organoids. Scale bar = 20 µm. e) Quantification of the percentage of acetylated tubulin positive ciliated cell (*n* = 4). f) qRT‐PCR analysis of PKD1, PKD2, and PKHD1 (ciliary genes), SLC34A1 and ATP1A1 (tubular epithelia transporter genes), ABCB1 and LRP2 (drug transporter genes), NPHS1, WT1, and PODXL (podocyte) and ECAD (distal tubule) (*n* = 3). g) Representative images of kidney organoids incubated fluorescence‐labeled (FITC) dextran 70 kDa and stained E‐cadherin (ECAD, distal tubule) and LTL (proximal tubule). h) Quantification of the percentage of FITC‐dextran 70 kDa positive cell. Scale bar = 20 µm (*n* = 3). Values are mean ± SEM. NS, no significance, *p < 0.05, **p < 0.01, ***p < 0.001, ****p < 0.0001, measured by one‐way ANOVA and two‐way ANOVA with Tukey's multiple comparisons test.

Progressive morphogenesis of tubular structures can be observed along with enhanced vascularization of the kidney organoids when they are transplanted into a mouse kidney, cultured in a microfluidic system, or implanted onto a chick chorioallantoic membrane.^[^ ^19^, ^20^, ^21^, ^22^, ^23^, ^24^ ^]^ Therefore, we hypothesized that progressive morphogenesis and maturation of gene expression profiles would occur in human iPSC‐derived tubular cells in kidney organoids in vitro when their vascularization was enhanced by the culture system based on kidney dECM. Enhanced polarization of the proximal tubules, with apical enrichment of the brush border marker lotus tetragonolobus lectin (LTL) and primary cilia, was observed in kidney organoids cultured by protocol A and B (**Figure** **4**a–e). Consistent with polarization, the expression of ciliary genes (PKD1, PKD2, PKHD1), genes of tubular epithelial transporters (SLC34A1, ATP1A1,), and genes of drug transporters (ABCB1, LRP2) as well as genes of podocytes (NPHS1, WT1, PODXL) and distal tubule (ECAD) was upregulated in kidney organoids cultured using protocol A and B (Figure 4f), indicative of enhanced functional potential (Figure 4f). We also performed an in vitro dextran uptake assay to examine whether kidney organoids exhibited physiologically relevant features (Figure 4g,h). The kidney organoids cultured by each of the four protocols were treated with 100 µg mL^−1^ of FITC dextran 70 kDa (Sigma‐Aldrich, 46945) at day 14. After 48 h of dextran treatment, tubular reabsorption of 70‐kDa dextran within LTL+ proximal tubule epithelial cells was extensively increased in kidney organoids generated by protocol A or B than in the Matrigel‐based or Matrigel + VEGF group (Figure 4g,h). Unfortunately, our method to treat dextran in the medium for culturing kidney organoids may not reflect the function of proximal tubules that reabsorb dextran from lumen to the outside of tubules. Direct microinjection into the tubular lumen would reveal the function of proximal tubules in kidney organoids. Therefore, our findings may be interpreted that 70‐kDa dextran leaking into lumen by immature barrier function of kidney organoids might be reabsorbed into LTL+ proximal tubule epithelial cells. Although the tubular function is still immature, we cautiously suggest that the resorption function of proximal tubular epithelial cells was enhanced in the protocol A or B organoid groups.

### 2.6 scRNAseq to Define the Effect of Kidney dECM on Kidney Organoid Development

**Figure 5:**
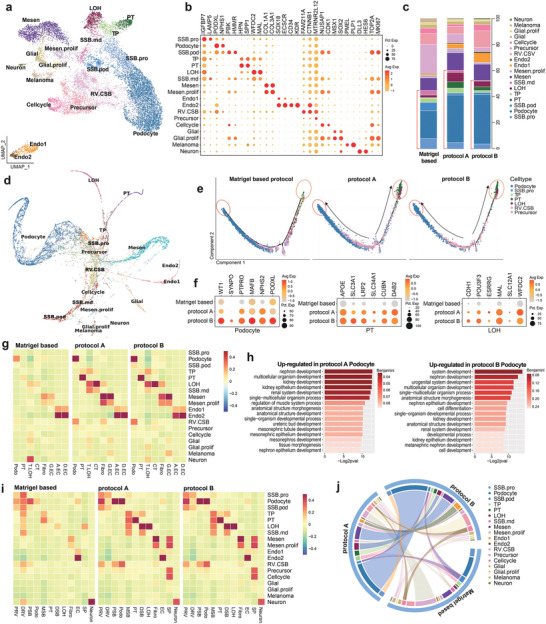
Single cell RNA‐seq analysis of kidney organoids. a) Uniform manifold approximation and projection (UMAP) embedding of analyzed single‐cell transcriptomes from 12 482 cells from kidney organoid cells annotated by cell type. b) Dot plot shows the expression of cell‐type‐specific genes. c) Each column corresponds to a single dataset. The stacked bar graph shows the cell type composition of each organoid from Matrigel‐based protocol, protocol A, and protocol B. d) The kidney organoid single‐cell transcriptome was reconstructed lineage progression by PAGA‐initialized ForceAtlas2 (via SCANPY). e) Pseudotime plots showing the distribution of each sample. The matured cells (podocyte, PT, and LOH) present strikingly distinct distribution to the end side of the trajectory. f) Dot plot comparing the expression of cell‐type signature and differentiation genes on Podocyte, PT, and LOH between the samples. g) Bar charts show GO enrichment terms of differentially expressed genes from protocol A and B podocytes. h,i) Heatmap of association of human kidney cell types and fetal kidney cell types with cell types in organoid samples, clustered by their expression of top cell‐type differentiating marker genes. j) Circos plot showing the similarity between samples by comparing each single‐cell expression datasets. Abbreviations: NP, nephron progenitor; P.prolif, Podocyte proliferate; TP, tubule progenitor; PT, proximal tubule; LOH, loop of Henle; T.prolif, tubule proliferate; Mesen, mesenchyme; Endo, Endothelium; T.LOH, Thick ascending limb of Loop of Henle; CT, connecting tubule; Fibro, Fibroblast; G.EC, glomerular endothelium; A.EC, ascending casa recta endothelium; D.EC, descending vasa recta endothelium; PRV, proximal renal vesicle; DRV, distal renal vesicle; PSB, proximal S‐shaped body; MSB, medial S‐shaped body; DSB, distal S‐shaped body; SP, stroma progenitor.

To test the effect of dECM on kidney organoid development at the single‐cell resolution, we performed scRNAseq for kidney organoid cells from three conditions; Matrigel‐based protocol, protocol A, and protocol B. After filtering out the cells by quality control and correcting for batch effects, 12 482 cells were visualized using the Uniform Manifold Approximation and Projection (UMAP). All cells were classified into 30 distinct clusters, and we annotated 18 cell types based on their unique transcript expressions (**Figure** **5**a). In Figure 5b, the dot‐plot shows the expression of marker genes across these clusters. We identified nephron cells; Podocyte (NPHS1+, PODXL+), Proximal tubule (HPN+, SPP1+), and Loop of Henle (WFDC2+, MAL+), along with a nephron progenitor cluster expressing a high level of IGFBP7, a progenitor marker, a tubule progenitor cluster expressing SPP1 and WFDC2, and a tubule marker. In addition, we identified Mesenchyme (COL1A1+), Endothelium (SOX18+, KDR+), proliferating cells (TOP2A+, MKI67+), and some off‐target cells; Glial (MSX1+, SOX2+), Melanoma (PMEL+, PLP1+), and Neuron (DLL3, HES6). We found two distinct sub‐clusters of endothelium cells: endothelium1 (SOX18+, ECSCR+) and endothelium2 (CD34+, KDR+). Moreover, we found precursor cells (MTRNR2L12+) and CTNNB1‐enriched cells that seem to be capable of differentiating into other cell types.

First, we checked cell‐type proportion in each organoid to analyze the effect of kidney dECM on kidney organoid differentiation into nephron cells. The results show that kidney dECM not only upregulates marker gene expression but increases the proportion of nephron cells in the kidney organoid (Figure 5c). In protocol A and B, podocytes made up 36.2% and 37.7% of the organoid. In Matrigel based protocol, however, podocytes made up only 21.2% of the organoid. Also, tubular epithelial cells (proximal tubule and LOH) made up 11.9% and 7.13% of organoid by protocol A and protocol B. In comparison, only 2.23% of organoid cells were differentiated into tubular epithelial cells by Matrigel‐based protocol. The proportion of off‐target cells were decreased by protocol A and B (Control, 7.75%; protocol A, 4.73%; protocol B, 5.08%). Also, proliferating cells (Control, 17.38%; protocol A, 9.1%; protocol B, 8.78%) and precursor cells and CTNNB1+ cells (Control, 30.18%; protocol A, 12.6%; protocol B, 15.84%) were decreased by protocol A and B; representing those organoid cells which were cultured on protocol A and B are processes in differentiation rather than proliferation.

Our data showed that kidney dECM increases the proportion of nephron cells in the organoid. Thus, we analyzed cellular differentiation and development connections in total organoid cells prior to comparing developmental differences among three samples. We used a force‐directed graph drawing algorithm, ForceAtlas2, to infer the differentiation trajectory of organoid cells. We initialized a ForceAtlas2 layout with partition‐based approximate graph abstraction (PAGA) coordinates from our annotated cell types. We obtained a topology tree graph that shows branching events from CTNNB1+ and precursor cells to independent differentiation branches for all other cell types (Figure 5d).

To verify the effect kidney dECM on kidney organoid maturation and discover developmental transition,^[^ ^25^, ^26^, ^27^ ^]^ we reconstructed kidney organoid cells by performing pseudo‐time ordering using Monocle2. The resulting cell trajectory revealed that the precursor cells are located at the branch point (middle) and differentiate into tubular epithelial cells and podocytes. Because the trajectory reflects cell maturity, the plots show that kidney organoid cells cultured on protocol A and B have more maturated cells (marked by circles in Figure 5e). Also, we confirmed that the expression level of cell‐type signature and differentiation‐related genes on podocyte, proximal tubule, and LOH are upregulated in protocol A and B (Figure 5f). Furthermore, we investigated upregulated genes in podocyte generated by protocol A and B. Functional enrichment analysis for GO biological process results in upregulated genes were mainly involved in kidney and nephron development (Figure 5g). These results represent that kidney dECM affects genes related to kidney development, resulting in improved maturating into nephron cells.

We used the CellCODE^[^ ^28^ ^]^ computational cell‐type deconvolution framework to investigate whether these kidney organoids show similarity to human kidney cells. We used marker genes of human kidney cell types and human fetal kidney cell types referred to Benjamin et al., and the estimated cell fraction values were used for heatmap (Figure 5h,i).^[^ ^29^ ^]^ Podocyte, PT, Mesenchyme, and Endothelial cells cultured on protocol A and B showed better similarity to the respective human kidney cell type (Figure 5h) as well as fetal kidney cell type (Figure 5i). Interestingly, Podocytes which induced by protocol A and B showed high similarity to human podocyte as well as fetal podocyte and PSB whereas Matrigel‐based protocol induced podocyte which had low similarity to these cells. Moreover, not only podocytes also another cell types that induced by protocol A and B also showed higher similarity to human and fetal kidney cell types (Figure 5i). Given the higher similarity to human kidney in protocol A and B, we compared those kidney organoids with each other using ClusterMap,^[^ ^30^ ^]^ which is designed to compare two or more single‐cell datasets. Data show that kidney organoids cultured on protocol A and B samples have high similarity to each other; however, kidney organoid grown on Matrigel‐based protocol was not (Figure 5j).

**Figure 6:**
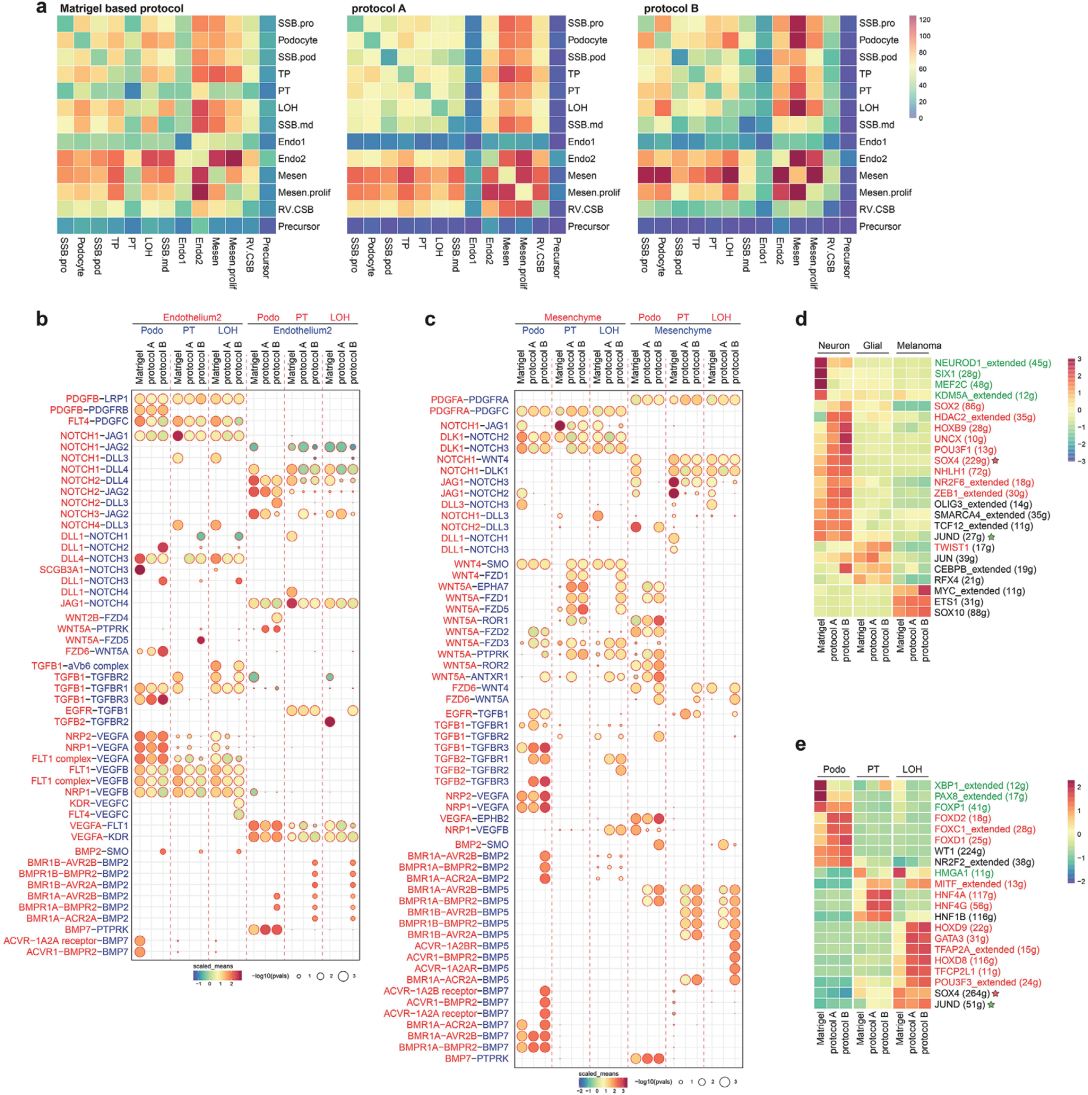
Cell communication network in kidney organoid. a) Heatmap showing the potential ligand‐receptor pairs between cell types predicted by CellphoneDB. Bubble plot showing the selected ligand‐receptor interactions with b) Endothelium2 or c) Mesenchyme cells; scaled means indicated by color and *p*‐value by circle size. Heatmap showing the scaled regulon activity from SCENIC in d) off‐target cell types and e) nephron cell types. The states of the transcription factors were indicated in red (activated in protocol A and B) and green (inactivated in protocol A and B). Abbreviations: SCENIC, single‐cell regulatory network inference and clustering.

Because our data showed that kidney dECM improves cell differentiation of kidney organoids, we analyzed which interactions between cell types play a vital role in kidney organoid differentiation in each sample. We used CellphoneDB to investigate the cell‐cell interaction networks among the cell types. Mesenchyme cells showed the highest interactions with endothelium2 in the case of Matrigel‐based protocol (**Figure** **6**a). And interactions with other cell types were higher in protocol A and B than in the Matrigel‐based protocol (Figure 6a). Kidney dECM is composed of essential growth factors, such as PDGF, TGF, and VEGF, and thus, we focused on those interactions between endothelium (or mesenchyme) and nephron cell types (Podocyte, Proximal tubule, and LOH) (Figure 6b,c). We also focused on multiple signaling pathways, such as Wnt, Bmp, and Notch, implicated in the proximal‐distal axis patterning of nephrons.^[^ ^31^, ^32^ ^]^

The VEGFs are key chemotactic factors that direct precursor cells into the glomerulus. Podocytes secrete VEGFA, which attracts endothelial precursors, while the glomerular endothelium releases PDGFB to attract mesangial cells.^[^ ^33^, ^34^ ^]^ Our data show that VEGFA interactions between mesenchyme cells and podocytes, which are essential for developing the mesangium and the glomerulus were more common in protocol A and B organoids than in the Matrigel‐based protocol.^[^ ^33^, ^35^, ^36^ ^]^

Notch‐mediated interactions, which play an essential role in early nephrogenesis but downregulates for differentiation toward podocyte lineage,^[^ ^37^ ^]^ were decreased in protocol A and B organoids. And TGFb1, expressed from endothelium and mesenchyme cells, showed increased interaction with TGFBR3, which is expressed on podocyte. BMP interactions with mesenchyme cells, one of the major factors in kidney development, was increased in protocol A and B organoids.^[^ ^38^, ^39^ ^]^ Especially, BMP2 and BMP7 expressed by podocyte, and BMP5 expressed by mesenchyme cells were significantly increased in kidney organoid cultured on protocol B. Taken together, the growth conditions that contains dECM enhanced cell–cell interactions, which enabled the kidney organoids to mature effectively.

To suggest which transcription factors might crucially affect the off‐target cell types, we posited that suppressing them during cultivation would effectively reduce the number of off‐target cells, so we analyzed the gene regulatory networks of the off‐target cell types using SCENIC (Figure 6d). We also analyzed the transcription factors essential for the nephron cell types to eliminate them from the candidate list (Figure 6e). According to our data, neuron cells were represented a tiny proportion of the cells in the kidney organoids, even though they were increased in protocol A and B organoids (0.16%, 0.55%, and 0.90%). Glial cells were present in equivalent amounts in every kidney organoid (2.8%, 2.2%, and 2.6%), whereas melanoma cells decreased in protocol A and B organoids (3.3%, 0.9%, and 0.8%). We found several transcription factors specific to off‐target cells, which might cause in inducing off‐target cells during organoid differentiation. For instance, the regulon activity of HDAC2, HOXB9, UNCX, and POU3F1 were specific for neuron cells while TWIST1, RFX4, and JUN regulon activity were specific for glial cells. Also, ETS1 and SOX10 were specific for melanoma cells. Comparing the TFs identified in kidney cell types, SOX4 and JUND should be excluded since they have an essential role in LOH formation. We suggest the rest of the transcription factors as potential candidates, which may reduce off‐target cells in kidney organoids by suppressing during cultivation.

In Figure 6d, we showed that several growth factors inhibit proper kidney organoid differentiation, such as that of TWIST1, which is specifically expressed in neuron and glial cells. Since TWIST1 was activated in neuron, we additionally analyzed the interactions that potentially enhance TWIST1. We found that the BMP signaling pathway, which serves as an upstream regulator of TWIST1, was increased in neuron and glial cells.^[^ ^40^, ^41^ ^]^ This mechanism likely occurs in a non‐cell‐autonomous fashion because the receptors of neurons and glial cells are highly affected by the BMP ligands secreted from their neighboring cells (other cell types) (Figure S7a,b, Supporting Information). Also, we assessed the expression level of TWIST1 regulons in neurons and found that some of the neuron development‐related genes, such as AXIN2, were increased in neurons grown using protocol A or B (Figure S7c,d, Supporting Information).^[^ ^42^ ^]^ So, we further tested whether TWIST1 inhibition affects suppression neurons, an off‐target cells, in kidney organoid differentiation. We administered 25 µm Harmine (Sigma‐Aldrich, 286044), a TWIST1 inhibitor, on day 15 of kidney organoid culture in the Matrigel‐based protocol for 24 h (Figure S7e,f, Supporting Information). Reduction in neuronal cells without alteration of the gross podocytes or tubular morphology was demonstrated by immunofluorescence analysis of an independent batch (Figure S7e, Supporting Information). qPCR data also showed a reduction in off‐target cell marker expression (Figure S7f, Supporting Information).

### 2.7 Recapitulation of Fabry Nephropathy with Vasculopathy Using Vascularized Kidney Organoids and CRISPR‐Cas9 Gene Editing

**Figure 7:**
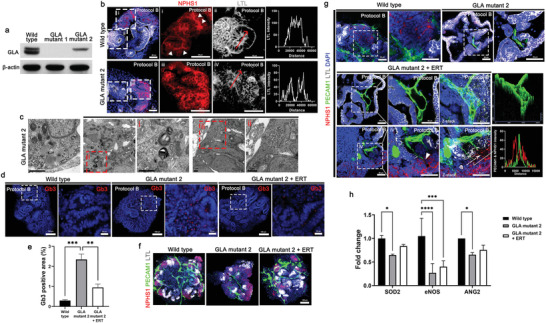
Recapitulation of Fabry nephropathy with vasculopathy using GLA‐mutant human kidney organoids differentiated by protocol B. a) Representative western blot for expression of GLA (clones GLA mutant 1 and GLA mutant 2) in kidney organoids. b) Representative image of immunofluorescent staining of NPHS1 and LTL in wild type and GLA‐mutant kidney organoid. LTL across the line scan indicated by red arrows. Scale bar = 50 µm. c) Representative TEM images showing the accumulation of lipid droplet, glycoprotein and zebra bodies. Scale bar = 1 µm. d) Representative images of immunofluorescent staining of Gb3 in wild type and GLA mutant kidney organoids treated human recombinant agalsidase‐*α* as an enzyme replacement therapy (ERT). Scale bar = 100 µm and 20 µm. e) Quantification of percentage of Gb3 positive area (*n* = 3). f,g) Representative images of immunofluorescent staining of NPHS1, PECAM1, and LTL in wild type and GLA‐mutant kidney organoids treated with ERT. 2.5D image and line scan (white arrow) obtained from Z‐stack confocal image. White arrowheads indicate the invasion of the PECAM1+ endothelial cells into NPHS1+ podocyte structure. Scale bar = 100 µm for (f) and Scale bar = 50 µm for (g). h) qRT‐PCR analysis of SOD2, eNOS, and ANG2 (*n* = 3). Values are mean ± SEM. *p < 0.05, **p < 0.01, ***p < 0.001, ****p < 0.0001, measured by one‐way ANOVA and two‐way ANOVA with Tukey's multiple comparisons test.

Fabry disease is a X‐linked inherited disorder that causes defects in the glycosphingolipid metabolic pathway that result from deficient or absent activity of the lysosomal enzyme, *α*‐galactosidase A (GLA), which lead to the accumulation of Gb3.^[^ ^43^, ^44^, ^45^ ^]^ In addition to Gb3 accumulation in the target cells, ischemia plays an important role in shaping the disease phenotype of Fabry disease such as cerebral vasculopathy, which is one of major cause of morbidity and early mortality in patients with Fabry disease.^[^ ^46^ ^]^ Furthermore, endothelial dysfunction is one of the mechanisms behind the development of end‐stage, irreversible complications of Fabry disease.^[^ ^46^ ^]^ Fabry nephropathy results from an accumulation of Gb3 in renal cells such as podocytes, glomerular endothelial cells, mesangial cells, tubular epithelial cells, and vascular endothelial cells, and it eventually causes end‐stage renal disease.^[^ ^44^, ^45^ ^]^ Vasculopathy in Fabry nephropathy has only partially been elucidated.^[^ ^43^, ^44^, ^45^, ^46^ ^]^ We generated two clones of GLA‐mutant human iPSCs (GLA‐mutant 1 and GLA‐mutant 2) by transfection with an all‐in‐one vector expressing Cas9, GLA‐specific single‐guide RNA, and GFP. Then the GLA‐mutant human iPSCs were differentiated into kidney organoids using protocol B. GLA proteins was absent in kidney organoids differentiated from GLA‐mutant 1 human iPSCs and decreased in kidney organoids differentiated from GLA‐mutant 2 human iPSCs (**Figure** **7**a). The structural deformity of kidney organoids differentiated from GLA‐mutant 1 human iPSCs was too severe to analyze their phenotypes, while those of kidney organoids differentiated from GLA‐mutant 2 human iPSCs was relatively mild to allow analysis. Therefore, we used kidney organoids differentiated from GLA‐mutant 2 human iPSCs as a model of Fabry disease in this study. The linear pattern of track with the NPHS1 expressed in basolateral aspect of podocytes was unorganized in GLA‐mutant kidney organoids (Figure 7b). Disruption of polarity of tubule was observe in GLA‐mutant kidney organoids (Figure 7b). Abundant lipid droplet, electron‐dense granular deposits and electron‐dense lamellate lipid‐like deposits that formed concentric bodies (zebra bodies) in the cytoplasm of podocytes and tubules was observed in GLA‐mutant kidney organoids (Figure 7c) which is compatible with phenotype of human renal Fabry disease. The accumulation of Gb3 is prominent in GLA‐mutant kidney organoids, which was decreased treated after recombinant enzyme replacement therapy (ERT) with agalsidase‐*α* (Figure 7d,e), These findings indicate that GLA‐mutant kidney organoids efficiently recapitulate Fabry disease. Next, we determine the phenotype of vasculopathy of Fabry disease using GLA‐mutant kidney organoids generated by protocol B. Vascularization was limited in GLA‐mutant human iPSC kidney organoids, while the wild type of kidney organoids was highly vascularized (Figure 7f,g). Interestingly, ERT with recombinant human agalsidase‐*α* in GLA‐mutant kidney organoids recovered the vascular network with glomerular vascularization and formation of large luminal vessels ((Figure 7f,g). The mRNA expression of the angiogenic factors, eNOS, and ANG2, and superoxide dismutase 2 (SOD2), mitochondrial antioxidant, were significantly decreased in GLA‐mutant kidney organoids, which is compatible with the phenotypes of vasculopathy in Fabry disease (Figure 7h).^[^ ^47^, ^48^ ^]^

Taken together, the data indicates that the methods generating vascularized kidney organoids combined with CRISPR‐Cas9 genome‐editing system are useful for disease modeling such as vasculopathy in Fabry disease and development therapeutic options.

### 2.8 Accelerated Formation of Vascular Network and Enhanced Maturation When Transplanted Kidney Organoids with Kidney dECM

Transplantation of human kidney organoids into living mouse kidneys enhances the formation of perfusable vasculature that facilitates the maturation of glomerular and tubular‐like structures in kidney organoids.^[^ ^19^, ^20^, ^21^, ^22^ ^]^ Considering that kidney dECM enhanced vascularization of kidney organoids in vitro, we hypothesized that transplantation of kidney organoids with kidney dECM could accelerated the vascularization into the transplanted graft, which could facilitate the morphogenesis of nephron‐like structures in the kidney organoids.

**Figure 8:**
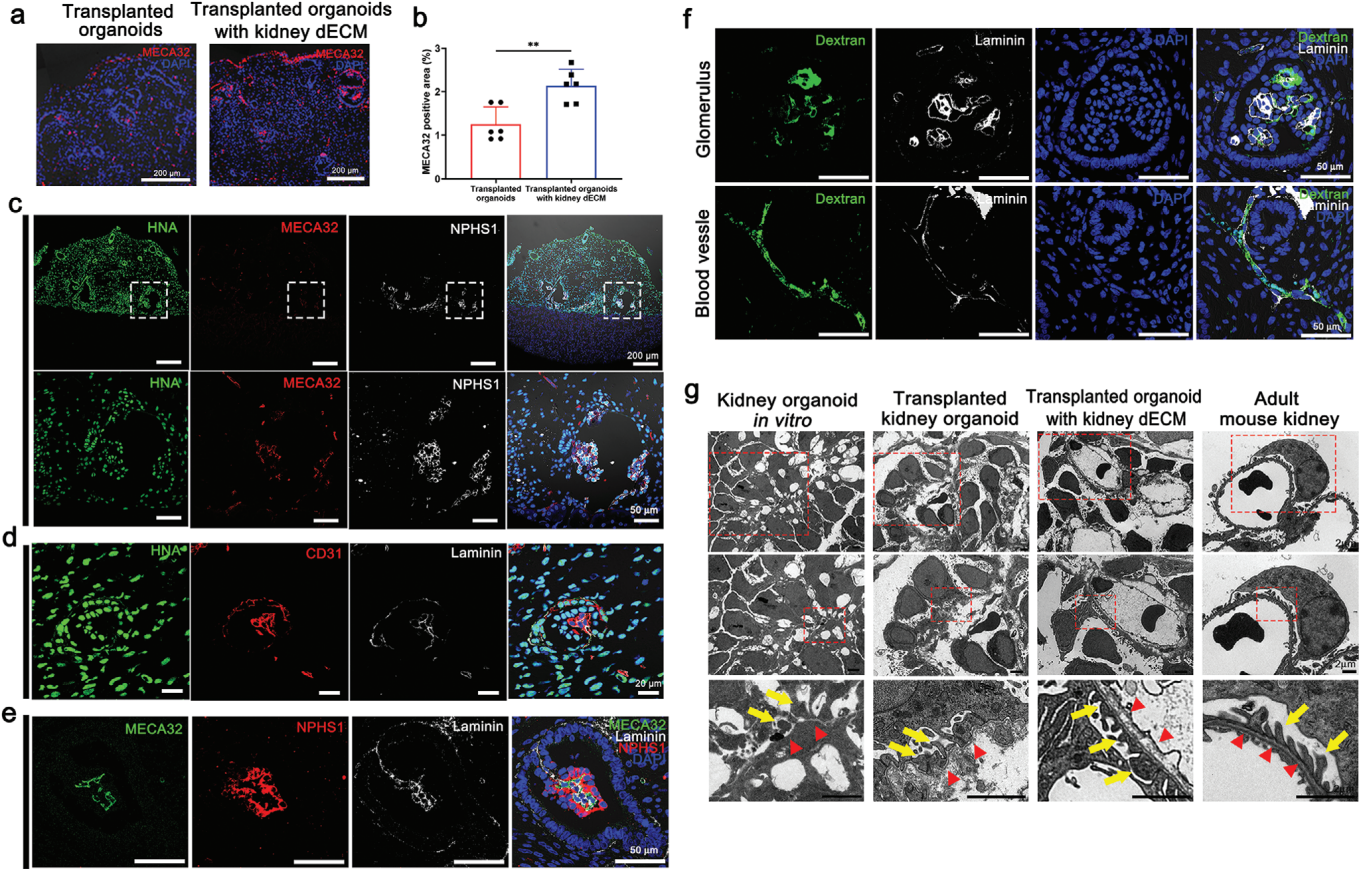
Accelerated formation of vascular network and maturation of glomerular‐like structures in kidney organoids in vivo when transplanted with kidney dECM. a,b) Representative confocal images of MECA32 in transplanted graft. MECA32‐positive cells were more abundantly observed in kidney organoids transplanted with kidney dECM, indicating the effect of kidney dECM in recruiting endothelial cells from the mouse kidney into the transplanted graft. Scale bar = 200 µm (*n* = 6). Values are mean ± SEM. **p < 0.01, measured by *t*‐test. c) Representative confocal images of HNA (Human nuclei antibody), MECA32 and NPHS1 in kidney organoids in vivo when transplanted with kidney dECM. d) Representative confocal images of HNA, CD31, and Laminin in kidney organoids in vivo when transplanted with kidney dECM. e) Representative confocal images of slit diaphragm like structures in kidney organoids transplanted with kidney dECM. Scale bar = 50 µm. f) Representative confocal images of fluorescein isothiocyanate (FITC)‐labeled dextran present inside the vessels and capillaries of glomerular‐like structures in the transplanted kidney organoids. Scale bar = 50 µm. g) Representative transmission electron microscopy (TEM) images of kidney organoids in vitro, transplanted kidney organoids, kidney organoids transplanted with kidney dECM, and adult mouse kidney. Red arrowheads indicated podocyte and tubule maturation structures. Scale bar = 2 µm.

To test this idea, we transplanted kidney organoids derived from human iPSCs with kidney dECM beneath the kidney capsule of immunodeficient NOD‐SCID mice for engraftment. During the following two weeks, mouse endothelial cells (MECA32+) were more abundantly observed in the transplanted kidney organoid graft with kidney dECM than in those without kidney dECM, indicating the effect of kidney dECM in recruiting endothelial cells from mouse kidney into the transplanted graft (**Figure** **8**a,b). Mouse endothelial cells (MECA32+) were abundantly observed in the transplanted kidney organoid graft as well as within the glomerulus‐like structures (Figure 8c), which indicates that endothelial cells from the host mouse kidney had extensively infiltrated into the transplanted kidney organoids and formed a vascular network. We also could observe human endothelial cells derived from the transplanted kidney organoids (CD31+/HNA+), but their proportion of the endothelial cells in the graft was small (Figure 8d). These findings indicated that the endothelial cells in the transplanted kidney organoids was not originated from endogenous endothelial progenitor cells but also the infiltrated mouse endothelial cells.^[^ ^6^ ^]^ The infiltrated mouse endothelial cells in glomerulus‐like structures lined with laminin, an extracellular matrix component of glomerular basement membrane (GBM) and NPHS1+ podocytes (Figure 8e). This suggested the formation of the filtration barrier interfacing among endothelial cells, GBM and podocytes in the grafts.

To study the integrity of the vasculature formed in the transplanted kidney organoids with kidney dECM, we injected 500 kDa of fluorescein isothiocyanate (FITC)‐labelled dextran into the tail vein of the mouse. FITC‐labelled dextran was present inside the vessels and capillaries of the glomerular‐like structures in the transplanted kidney organoids (Figure 8f), which suggests that the vasculature in the transplanted kidney organoids connected to the host mouse‐derived kidney vasculature and thereby maintained its vascular integrity.

We further analyzed these structures by transmission electron microscopy (TEM) to determine the cellular ultrastructure, comparing the kidney organoids in vitro, transplanted kidney organoids, and adult mouse kidney. When transplanted kidney organoids without kidney dECM, the podocytes looked similar to the organoids in vitro and direct comparison to the adult mouse kidney demonstrated that organoids lacked bona fide foot processes with well‐organized tertiary interdigitations along the GBM (Figure 8g). Glomerulus‐like structures were surrounded by a tubular epithelium with intervening space, similar in architecture to a Bowman's capsule but with a substantially thicker capsular layer (Figure 8g). In the kidney organoids in vitro, the podocytes were arranged intermittently along poorly organized GBM‐like tracks (Figure 8e). When we examined the transplanted kidney organoids without kidney dECM, red blood cell fragment was observed capillary lumen‐like structure, which suggested possible capillary formation (Figure 8g). Although the GBM was formed in the transplanted graft, its thickness was irregular and thicker than in the adult mouse kidney (Figure 8g). In the transplanted kidney organoids with kidney dECM, GBM was well organized with alignment with the podocyte and endothelial cells and the podocytes had secondary or tertiary foot process interdigitated with GBM similar to those of adult mouse kidney, similar to the findings on the adult mouse kidneys (Figure 8g, arrow heads)

Taken together, our findings suggest that kidney dECM accelerated the recruitment of endothelial cells from the host mouse kidney, formed a vascular network, maintained vascular integrity, and contributed to the maturation of glomerular‐like structures in the transplanted kidney organoids.

## 3 Discussion

Human kidneys are highly vascularized organs that normally receive approximately 20% of the cardiac output.^[^ ^49^ ^]^ Considering the lack of vasculature and structural insufficiency of kidney organoids, developing an appropriate renal vasculature and spatial arrangement and enabling interactions with corresponding nephrons are crucial for the generation of a functioning kidney.^[^ ^49^ ^]^ There are some challenges to overcome lack of vascularization in kidney organoid culture. Czerniecki et al. increased the vascularization of kidney organoids through addition of exogenous VEGF during the differentiation process.^[^ ^50^ ^]^ Low et al. enhanced the formation of a vascular network by precise modulation of WNT signaling.^[^ ^51^ ^]^ However, the maturation of a nephron‐like structure with endothelial invasion into the glomerulus in kidney organoids in vitro was unclear. Transplantation of kidney organoids onto chick chorioallantoic membrane or mouse kidney under the renal capsule enhanced vascularization of kidney organoids and maturation of nephrons with endothelial invasion into glomeruli of transplanted kidney organoids.^[^ ^20^, ^23^ ^]^

We focused on the physiologic role of ECM in kidney organogenesis, including mesenchymal condensation, nephrogenesis, terminal differentiation of renal tubules, and GBM assembly. The kidney dECM based methodologies described here generated highly vascularized human iPSC‐derived kidney organoids in vitro, with invasion of endothelial cells into a glomerulus‐like structure and greater maturation of cellular compartments than found with the classical protocol. The strength of our protocol based on the kidney was achievement of vascularization and maturation of kidney organoids in vitro without transplantation. The highly vascularized kidney organoids can provide a reliable platform for investigating kidney diseases such as diabetic nephropathy having microvascular complications. Methodologies enhancing maturation without transplantation can facilitate clinical use of kidney organoids.

Homan et al. also induced substantial vascularization and morphological maturation in kidney organoids in vitro using flow on millifluidic chips.^[^ ^24^ ^]^ Their method showed that vascularized kidney organoids cultured under flow, especially high flow, had more mature podocytes and tubular compartments, with enhanced cellular polarity and adult gene expression, than did static controls.^[^ ^24^ ^]^ Application of a microfluidic system with our kidney dECM based methodologies is a future challenge to improve the vascularization and maturation of kidney organoids. Furthermore, application bioengineering and 3D bioprinting technologies can engineer kidney organoids to form a pattern, differentiate, and morpho‐genesis into more realistic “kidney in a dish.”

Single cell RNA‐seq showed that ECM enhanced cell–cell interactions within the kidney organoids through the actions of their own growth factors and cytokines, producing mature cell clusters within the kidney organoids. The kidney dECM based methodology for the generation of kidney organoids can optimally recapitulate kidney development and provide tools for understanding the mechanisms of ECM that regulate kidney organogenesis.

In this study, the concentration of VEGF was increased in kidney dECM (Table S1, Supporting Information). Considering the VEGF enhances vascularization,^[^ ^50^ ^]^ we determined the effect of kidney dECM on vascularization by comparing a Matrigel+VEGF group as a control with groups using protocol A or B (Figure 3a). VEGF enhanced vascularization of kidney organoids, but vascularization was less widespread than those of protocols A and B. Furthermore, VEGF alone did not cause PECAM1+ cells to invade the glomerulus, while PECAM1+ cells invaded the glomerulus in protocol A; this invasion was more prominent when VEGF was added on kidney dECM (Protocol B) (Figure 3a).

To clarify the effect of VEGF on vascularization of kidney organoids differentiated by protocol A or B, we inhibited VEGF with bevacizumab (Figure S8a,b, Supporting Information). Kidney organoids cultured in each of the four protocols were treated with 25 µg mL^−1^ of bevacizumab (Selleckchem, A2006), a VEGF inhibitor, every 3 days from day 7 to day 18. The VEGF inhibition resulted in loss of PECAM1+ endothelial cells in kidney organoids differentiated by the Matrigel‐based protocol or Matrigel+ VEGF protocol (Figure S8a,b, Supporting Information). Notably, although a significant decrease in the distribution of PECAM1+ endothelial cells by VEGF inhibition was observed with protocols A and B, its presence persisted (Figure S5a,b, Supporting Information). Taken together, these findings suggest an effect other than that of VEGF of kidney dECM on vascularization although kidney dECM contains a higher concentration of VEGF.

Furthermore, single‐cell RNA sequencing analysis showed that VEGF interaction was increased by additional VEGF on kidney dECM (protocol B). On the other hand, the VEGF interaction was showed no change in pattern with protocol A (Figure 6b). These findings suggest that adding VEGF on kidney dECM enhanced the vascularization by increasing the interaction of kidney dECM compositions, not only that of VEGF, but also those of TGFB, PDGF, and other growth factors.

Another interesting point of this study is the recapitulation of Fabry nephropathy with vasculopathy by combining our kidney dECM–based methodology for generating vascularized kidney organoids with CRISPR‐Cas9 gene editing of GLA. Interestingly, after ERT, the vascularized kidney organoid recapitulating Fabry nephropathy formed a microvasculature that fenestrated into podocytes and surrounded the tubular structures, recovering the structural changes in the podocytes and tubular cells. Thus, the hPSC‐derived vascularized kidney organoids modeling Fabry disease could be used to investigate the mechanism of Fabry nephropathy and develop therapeutic tools. Our findings also suggest that combining vascularized kidney organoids with CRISPR‐Cas9 gene editing can be used in kidney disease modeling, especially for diseases accompanied by vasculopathy or endothelial dysfunction, such as diabetic nephropathy. Those disease models could then be used for nephrotoxicity testing of drugs that cause injury to renal endothelial cells.

In our previous study, we reported that kidney organoids transplanted beneath the kidney capsules of immunodeficient mice became vascularized from host mouse endothelial cells and that the nephron‐like structures in the grafts more matured than kidney organoids in vitro. However, the transplanted kidney organoids remained immature compared with the neighboring mouse kidney tissue.^[^ ^6^ ^]^ Immaturity was a common feature of three separate classical differentiation protocols for kidney organoids.^[^ ^52^ ^]^

We transplanted kidney organoids with kidney dECM hydrogels, which resulted in robust angiogenesis sprouting from the existing blood vessels of host mouse kidney, maintained their vascular integrity, and enhance the maturation of glomerular‐like structures. We envisage that kidney dECM‐derived hydrogels could be used for future kidney organoid transplantation and bioprinting in clinically relevant environments

In conclusion, the ECM‐based methodologies described here are broadly applicable and adaptable to other organoid types, which will enhance organoid differentiation both in vitro and in vivo for use in experimental investigations of the molecular pathways of human development and diseases or regenerative medicine.

## 4 Experimental Section

### Decellularization of Kidney

Fresh porcine kidneys were first prepared by removing the renal capsule and perirenal fat. A sagittal section of the kidney was prepared through removal of intrarenal fat and the renal medulla. The remaining cortex tissue was frozen, sliced into 0.1−0.3‐mm slices, and washed three times with distilled water for 30 min. Next, the slices were treated with 0.5% Triton X‐100 (Sigma‐Aldrich) in 1 m NaCl (Samchun Pure Chemicals) for 16 h. Then they were washed three more times for 1 h. DNase was administered at 37 °C for 6–7 h to eliminate any remaining cellular components. The Dnase‐treated tissue slices were then washed with PBS for 12 h, followed by sterilization with a 0.1% peracetic acid solution for 1 h and another washing with distilled water. The decellularized tissues were freeze‐dried at −80 °C and then used for biochemical characterization and kidney dECM hydrogel preparation.

### Preparation of Kidney dECM Solution

To prepare kidney dECM solution, 10 mg of lyophilized kidney dECM tissue was dissolved per 1 mL of 0.5 m acetic acid. Then, 1 mg of pepsin (Sigma‐Aldrich) was added for digestion in every 10 mg of lyophilized kidney tissue. The mixture was prepared in a 50 mL conical tube, and a magnet was included for stirring on a stirrer plate for 24 h, the estimated time required to fully dissolve kdECM solution.

### Oscillatory Rheology

Rheological properties of Matrigel and kidney dECM pre‐gel were investigated using a discovery hybrid rheometer‐2 (TA Instruments). Temperature ramping oscillatory test was performed to evaluate the gelation kinetics of kidney dECM pre‐gel by measuring its storage (*G*′) and loss modulus (*G*″). Respective samples of neutralized kidney dECM pre‐gel and Matrigel were loaded onto the Peltier plate at a volume of 150 µL. Measurements were taken using a steel 20 mm parallel plate geometry with gap size of 600 µm with temperature range from 20 to 37 °C at a heating rate of 5 °C min^−1^, and a constant frequency of 1.59 Hz was applied over 2000 s duration. All tests were performed in triplicate.

### Scanning Electron Microscopy (SEM)

SEM images of kidney dECM hydrogel were taken to examine its microstructural topography. Samples were fixed using 2.5% glutaraldehyde (G5882, Sigma) followed by phosphate buffer saline washing. The washed samples were then rinsed three times in deionized water to remove remnant salt. Specimens were then dehydrated in series of increasing ethanol gradient until treatment using 100% ethanol (50%, 60%, 70%, 80%, 90%, 95%, and 100%).

Specimens were then coated with Pt at 10 nm thickness using a sputter coater (EM ACE200, Leica) and loaded onto a field‐emission scanning electron microscope (Sigma, Carl Zeiss). The loaded specimens were then observed, and images were retrieved using smart SEM software.

### Biochemical Characterization of Kidney dECM

To quantify double stranded DNA (dsDNA), the kidney dECM was digested in 1 mL of papain solution (125 µg mL^−1^ papain in 0.1 m sodium phosphate with 5 mm Na2‐EDTA and 5 mm cysteine‐HCl at pH 6.5) for 16 h at 60 °C. Then, the dsDNA was isolated from the digested sample using a GeneJET Genomic DNA Purification Kit (Thermo Scientific, USA). Next, 1 µL of the digested samples was loaded on a NanoDrop (Thermo Scientific), and its contents were measured. For the immunohistochemical analysis, native kidney and decellularized tissues were fixed in 10% formalin, embedded in paraffin, and sectioned with a microtome. The sectioned samples were stained with H&E, Alcian blue, Masson's trichrome, and anti‐fibronectin. The stained samples were then examined with a light microscope.

### LC‐MS/MS Analysis

Tissues were cryo‐pulverized in liquid nitrogen using Covaris Tissue CryoPrep system (Covaris, Woburn, MA, USA). The powdered samples were collected and placed in glass tubes. The protein pellets were suspended in 1 mL of lysis buffer (8 m urea–0.1 m Tris‐HCl buffer, pH 8.5) and 40 µL of protease inhibitor cocktail (25× stock), followed by sonication for 20–40 min at 15 °C using a Covaris S2 Focused‐Ultrasonicator (Covaris, Woburn, MA, USA). The concentration of protein was quantified using the Pierce BCA Protein Assay Kit (Thermo Fisher Scientific). The digestion step was performed using FASP on a Microcon 30K centrifugal filter device (Millipore, Billerica, MA, USA). Each 100 µg of protein sample was reduced by incubating with TCEP at 37 °C for 30 min and alkylated with IAA at 25 °C for 1 h in dark conditions. After washing with lysis buffer and 50 mm ABC sequentially, the proteins were digested with trypsin (enzyme to protein ratio of 1:50; w/w) at 37 °C for 18 h. The resulting peptide mixtures were transferred to new tubes, and trypsin was inactivated by acidifying with 15 µL of formic acid. The digested peptides were desalted using C18 spin columns (Harvard Apparatus, Holliston, MA, USA), and the peptides were eluted with 80% acetonitrile in 0.1% formic acid (Honeywell, Charlotte, NC, USA) in distilled water.

The prepared samples were resuspended in 0.1% formic acid in water and analyzed using a Q‐Exactive Orbitrap hybrid mass spectrometer (Thermo Fisher Scientific, Waltham, MA, USA) along with an Ultimate 3000 system (Thermo Fisher Scientific, Waltham, MA, USA). Depending on peptide hydrophobicity, a 2 cm × 75 µm ID trap column packed with 3 µm C18 resin or a 50 cm × 75 µm ID analytical column packed with 2 µm C18 resin was used. A data‐dependent acquisition method was adopted, and the top 10 precursor peaks were selected and isolated for fragmentation. Ions were scanned at high resolution (70 000 in MS1, 17 500 in MS2 at *m*/*z* 400), and the MS scan range was 400–2000 *m*/*z* at both the MS1 and MS2 levels. Precursor ions were fragmented with NCE (Normalized Collisional Energy) 27%. Dynamic exclusion was set to 30 s.

### Proteome Data Analysis

Thermo MS/MS raw files of each analysis were searched using Proteome Discoverer software (ver. 2.5), and the Sus scrofa database was downloaded from Uniprot. The appropriate consensus workflow included a peptide‐spectrum match (PSM) validation step and SEQUEST HT process for detection as a database search algorithm. The search parameters were set up as follows: 10 ppm of tolerance of precursor ion masses, 0.02 Da fragment ion mass, and maximum of two missed cleavages with trypsin enzyme. Dynamic modification of the peptide sequence was as follows: static carbamidomethylation of cysteine (+57.012 Da), variable modifications of methionine oxidation (+15.995 Da), acetylation of protein N‐term (+42.011 Da), and carbamylation of protein N‐term (+43.0006 Da). After searching, results below 1% of FDR were selected.

### Kidney Organoid Differentiation

The WTC11 and CMC11 iPSC cell line (male donor) obtained from The Catholic University of Korea were used. Cells were used between passages 30 and 60. Kidney organoid differentiation without kidney dECM was performed as described previously (1). For Matrigel‐based protocol, hPSCs were plated at a density of 5000 cells/well of a 24‐well plate in mTeSR1 medium (Stem Cell Technologies) + 10 µm Y27632 (LC Laboratories) on glass plates (LabTek) coated with 0.1% GelTrex (Thermo Fisher Scientific) (day 1). The medium was exchanged for 1.5% GelTrex in mTeSR1 (day 2), mTeSR1 (day 3), RPMI (Thermo Fisher Scientific) + 11 µm CHIR99021 (Tocris) (day 4), or RPMI + B27 supplement (Thermo Fisher Scientific) (day 5) and cells were fed every 2–3 days to promote kidney organoid differentiation.

The Matrigel + VEGF Protocol Was the Same as the Matrigel‐Based Protocol but with Addition of VEGF on Days 6, 9, 12, and 14

For the kidney organoids differentiation based on kidney dECM, two protocols were developed. Two protocols were followed Freedman's protocol and modification was made as follow.^[^ ^1^ ^]^ In protocol A, each well of a 24‐well plate was coated with diluted kidney dECM (0.1%) and then dissociated, undifferentiated human iPSCs were placed evenly. After 24 h, by adding 1.5% Matrigel, human iPSCs were sandwiched between a lower layer of kidney dECM and an upper layer of Matrigel.

On day 4, CHIR was administered to the cells, which were fed every 2–3 days with an appropriate medium, as described in Figure 2a, to promote kidney organoid differentiation. Protocol B followed protocol A, but VEGF was added on days 6, 9, 12, and 14 to enhance the vascular network and SB‐431542 on day 12 to enhance podocyte differentiation.

### Immunofluorescent Analyses

For immunofluorescence, organoids were fixed on day 18 unless otherwise noted. For fixation, an equal volume of PBS (Thermo Fisher Scientific) + 8% paraformaldehyde (Electron Microscopy Sciences) was added to the medium for 15 min, after which the samples were washed three times with PBS. The fixed organoid cultures were blocked in 5% donkey serum (Millipore) + 0.3% Triton‐X‐100/PBS, incubated overnight in 3% bovine serum albumin (Sigma) + PBS with primary antibodies, washed, incubated with AlexaFluor secondary antibodies (Invitrogen), washed, and stained with DAPI or mounted in Vectashield H‐1000. Images were acquired using a Zeiss LSM 700 confocal microscope (Carl Zeiss, Germany) and ZEN 3.1 software.

The following primary antibodies were used: anti‐Acetylated tubulin (Sigma‐Aldrich T7451, 1:200), anti‐CD31 (R&D AF3628, 1:200), anti‐Collagen I (Southern Biotech 1310‐01, 1:200), anti‐Collagen IV (Southern Biotech 1340‐01, 1:300), anti‐ E Cadherin (Abcam ab11512, 1:100), anti‐Gb3 (TCI A2506, 1:200), anti‐GLA (Thermo Fisher Scientific PA5‐27349, 1:1000), anti‐Nuclei antibody (Millipore MAB 1281, 1:200), anti‐Laminin (Sigma‐Aldrich L9393. 1:200), anti‐LTL (Vector Labs FL‐1321, 1:200 dilution), anti‐MAP2 (Sigma‐Aldrich M4403, 1:200), anti‐MECA32 (BD Pharmingen 555849, 1:200), anti‐NPHS1 (R&D AF4269, 1:200), anti‐Pecam1 (Abcam ab9498, 1:200), anti‐TUBA4A (Abcam ab24610, 1:200), and anti‐WT1 (Abcam ab89901, 1:200).

### Real‐Time Quantitative PCR Analysis

Kidney organoid samples were harvested and total RNA was isolated using the RNAiso Plus Kit (Takara, Japan). Complementary DNA was synthesized using a Maxima First Strand cDNA Synthesis Kit for RT‐qPCR (Thermo Fisher Scientific, USA). Gene expression was analyzed with Power SYBR Green PCR Master Mix (Applied Biosystems, USA) using real‐time PCR (Applied Biosystems, Foster City, CA). qRT‐PCR were performed in triplicate and the relative mRNA expression levels were determined using the 2‐ΔΔCt method. The primer sequences used are in Table S4, Supporting Information.

### Western Blot Analysis

The kidney organoids were homogenized in lysis buffer (1% SDS, 1 mm sodium orthovanadate, and 10 mm Tris, pH 7.4). The protein concentration was determined by the BCA Protein assay kit (Thermo Fisher Scientific). Equal amounts of protein were separated on SDS–polyacrylamide gel and transferred to an NC membrane. For immunodetection, the blots were incubated overnight in PBS containing 5% skim milk and 0.1% Tween‐20 with the primary antibody. The blots were washed several times and then incubated with secondary antibody conjugated to horseradish peroxidase (Jackson ImmunoResearch Laboratories). The blots were visualized using a western blotting luminol reagent kit (Santa Cruz Biotechnology, Santa Cruz, CA).

### Transmission Electron Microscopy (TEM) Analysis

Adult mouse kidney block samples, samples from the transplanted kidney organoids, and kidney organoid in vitro samples were fixed in 4% paraformaldehyde and 2.5% glutaraldehyde in 0.1 m phosphate buffer overnight at 4 °C. After washing in 0.1 m phosphate buffer, the samples were postfixed with 1% osmium tetroxide in the same buffer for 1 h at 4 °C. Next, the samples were dehydrated with a series of graded ethyl alcohol solutions, exchanged through acetone, and embedded in Epon 812.

Ultrathin sections (70–80 nm) were obtained using an ultramicrotome (Leica Ultracut UCT, Germany). The ultrathin sections were double‐stained with uranyl acetate and lead citrate, after which they were examined under a TEM (JEM 1010, Japan) at 60 kV. For quantitative analysis, 20 low‐magnification (×6000) fields were randomly selected from each section of the cortex, and the number of autophagosomes per 100 µm^2^ was determined.

For the correlative light‐ and electron‐microscopic study, vibratome sections were cryoprotected with 2.3 m sucrose in 0.1 MPB and frozen in liquid nitrogen. Semi‐thin cryosections (2‐µm thick) were cut at −100 °C with a glass knife in a Leica EM UC7 ultramicrotome equipped with an FC7 cryochamber (Leica). The sections were labeled at 4 °C overnight using a mouse polyclonal antibody against PCAM1 and WT1. Antibody staining was visualized using Alexa Fluor secondary antibodies (Invitrogen). Sections were counterstained with DAPI for 10 min. Coverslipped sections were examined with a confocal microscope and photographed at ×200 or ×400 magnification with a differential interference contrast setting to find specific areas for later examination by electron microscopy. After the coverslips were floated off the sections, silver enhancement was performed using an HQ silver enhancement kit (Nanoprobes) for 3 min, and the tissues were prepared for EM as described previously.^[^ ^53^, ^54^ ^]^

### Generation of GLA‐KO Human iPSCs and Kidney Organoids

Generation of GLA‐KO human iPSCs was described previously.^[^ ^55^ ^]^ In brief, human GLA‐specific gRNA sequence was provided by Invitrogen Life Technologies (GLA gRNA sequence: TTGGCAAGGACGCCTACCAT). Oligo annealing and subcloning into Cas9 nuclease reporter vector were performed according to the manufacturer's instructions. All‐in‐one Cas9 nuclease reporter vector expressing Cas9 including gRNA against GLA and GFP was transfected by electroporation into iPSCs and then incubated for 7–10 days. GFP‐expressing cells were separated using FACS, seeded on 96‐well plates as single cells, and incubated until reaching pure clone state. A total of 6 clones expressing GFP was obtained and analyzed using Sanger sequencing. Two of 6 clones, GLA mutant 1 and GLA mutant 2, were identified to harbor alteration at the genetic lesion targeted using the GLA‐specific sgRNA‐mediated CRISPR/Cas9 and were used for western blot analysis. Kidney organoids were differentiated from GLA mutant 1 and GLA mutant 2 human iPSCs. To determine the effect of ERT, the enzyme rh*α*‐GLA was applied to the GLA‐mutant kidney organoids at doses of 9 µg starting on day 15 for 3 days at 5% CO_2_ in an incubator.

### Transplantation of Kidney Organoids Derived from Human iPSCs

Adherent organoids were micro‐dissected from 24‐well plates on day 18 of differentiation with a 23‐gauge syringe needle, after which they were carefully transferred using a transfer pipette into an Eppendorf tube containing RPMI + B27 supplement. The harvested kidney organoids were transplanted with 0.1% kidney dECM into the renal subcapsular space of eight‐week‐old immunodeficient male NOD/SCID mice (Jackson Laboratories, West Grove, PA, USA). In brief, the mice were anesthetized with zoletil, after which the kidney was exposed via a dorsal flank incision. After incision of the host kidney capsule (approximately 2 mm) with a 23‐gauge syringe needle, a PE50 tube containing 10–20 kidney organoids was carefully placed under the kidney capsule. The kidney organoids and 0.1% kidney dECM were delivered by carefully blowing through the other side of the PE50 tube. Mice were sacrificed 14 days after transplantation (*n* = 3 per group). All surgical interventions and presurgical and postsurgical animal care were provided in accordance with the Laboratory Animals Welfare Act, the Guide for the Care and Use of Laboratory Animals and the Guidelines and Policies for Rodent Survival Surgery provided by the IACUC (Institutional Animal Care and Use Committee) in School of Medicine, The Catholic University of Korea (Approval number: CUMS‐2020‐0171‐01).

### Processing and Analysis of Single‐Cell RNA seq

The quality control filtering of cells and genes was performed using only cells having at least 800 detected transcripts; cells with zero and more than 15 mtDNA read counts were excluded. To eliminate potential doublets, single cells with more than 5000 genes detected were filtered. Finally, 12 482 single cells remained and were processed with Seurat R package (v.3.2.3). To avoid a batch effect, the method fastMNN was used to detect mutual nearest neighbors (MNN) of cells in different batches.

Total kidney organoid cells were classified into 30 distinct clusters and merged based on differentially expressed genes (10<DEGs). Next, 18 clusters were annotated based on conventional markers used to categorize cell types into known biological types. The conventional markers are shown in Table S5, Supporting Information. Using the Seurat R package, unwanted cells were filtered out based on their mitochondrial gene representation and variances in unique gene counts. Downstream analysis was performed using the Seurat package following UMAP dimensional reduction. The single cells were clustered using the FindNeighbors (using 30 PCs for dimension reduction) and FindClusters functions (resolution = 1) in the Seurat (v.3.2.3) package.

### Trajectory Analysis

Monocle2 (v.2.14.0) was used, which learns a trajectory graph from a dimension reduction, to discover developmental transitions. Highly variable genes identified by Seurat were used to sort cells in pseudo‐time order. Then the data was reduced dimensionality and ordered cells along the trajectory for visualization. Cells were plotted using the “plot_cell_trajectory” visualization function.

For lineage tree reconstruction, PAGA was used, which quantifies the connectivity between clusters of cells and generates a graph representing the trajectories observed during reprogramming. The PAGA algorithm was performed using the scanpy.tl.paga function in the Scanpy package (v.1.6.0), with the Seurat cell clusters as input.

### GO Enrichment Analysis

The functional enrichment analysis of differentially expressed genes was performed using DAVID online tools (version DAVID 6.8; https://david.ncifcrf.gov/).

### Cell–Cell Communication Analysis

CellphoneDB is a Python‐based computational analysis tool developed by Roser Vento‐Tormo et al.,^[^ ^56^ ^]^ that can analyze cell–cell communication at the molecular level. Interaction pairs which are the PDGF, NOTCH, WNT, TGFB, VEGF, and BMP families, were selected to evaluate the relationships among cell types. The “plot_cpdb” function was used in the ktplots package (https://github.com/zktuong/ktplots) to visualize the dot‐plot.

### Single‐Cell Regulatory Network Analysis

SCENIC is a computational method used to construct regulatory networks and identify different cell states. For the analysis, the hg19 motif ranking database supplied in the SCENIC package was used to score potential regulons. To analyze the difference among cell types based on transcription factors, the Limma package calculated preferentially expressed regulons.

### Statistical Analysis

For all quantitative measurements, the entire population was used to calculate statistical significance, whereas mean values with *n* = 3 were used to calculate standard error and graphical confidence intervals. Data were then analyzed using Mann–Whitney tests or Kruskal–Wallis test to determine significance among groups. On each graph, the error bars represent −2 SEM (standard error of the mean), a 95% confidence interval. A single asterisk was used for *p*‐values < 0.05, two asterisks for *p* < 0.01, and three asterisks for *p* < 0.001.

## Conflict of Interest

The authors declare no conflict of interest.

## Author Contributions

J.W.K., S.A.N., J.Y., and J.Y.K. contributed equally to this work. J.W.K., S.A.N., J.Y., J.Y.K., Y.C., J.Y.L., and H.L.K. contributed to conducting experiments. J.P., D.W.C., and Y.K.K. contributed to designing research and writing paper.

## Supporting information

Supporting Information

## Data Availability

The data that support the findings of this study are available in the supplementary material of this article.
