# Supplementary material for: Kidney Decellularized Extracellular Matrix Enhanced the Vascularization and Maturation of Human Kidney Organoids
Source: Adv Sci (Weinh). 2022 Mar 24;9(15):2103526. doi: 10.1002/advs.202103526 (PMC9130892; doi:10.1002/advs.202103526)
Supplement: Supplementary file 1 — Supporting Information [file ADVS-9-2103526-s001.pdf]

Supporting Information

Kidney decellularized extracellular matrix enhanced the vascularization and maturation of human kidney organoids

Jin Won Kim,[1] Sun Ah Nam,[1]* Jawoon Yi,[2]* Jae Yun Kim,[3]* Jong Young Lee,[1] Seo-Yeon Park,[1] Tugce Sen,[4] Yoo-mi Choi,[5] Jae Yeon Lee,[6] Hong Lim Kim,[7] Hyung Wook Kim,[8] Jiwhan Park,[2]* Dong-Woo Cho,[3,4]* Yong Kyun Kim[1,8]**

Table S1. Growth factor and cytokine concentrations (pg/ml) in kidney dECM and native kidney samples

	**Kidney dECM**	**Native kidney**
Basic fibroblast growth factor (bFGF)	25.63	255.3
Endothelial growth factor (EG-VEGF)	27.65	486.3
Glial cell-derived neurotrophic factor (GDNF)	2.658	32.65
Growth hormone (GH)	110.23	2635.3
Insulin-like growth factor-binding protein 1(IGFBP-1)	63.25	785.3
Insulin-like growth factor-binding protein 2(IGFBP-2)	42.5	893.2
Insulin-like growth factor-binding protein 4(IGFBP-4)	2221.3	4555.1
Insulin-like growth factor-binding protein 6(IGFBP-6)	203.12	6826.3
Insulin	489.33	4852.6
Platelet-derived growth factor receptor AA(PDGF-AA)	80.69	35.69
Placental growth factor (PIGF)	6.39	41.32
Transforming growth factor alpha (TGF-α)	53.36	598.3
Transforming growth factor beta 1 (TGF-β 1)	5625.3	24545.6
Transforming growth factor beta 3 (TGF-β 3)	186.63	523.63
Vascular endothelial growth factor (VEGF)	652.36	156.39
Vascular endothelial growth factor R2 (VEGF R2)	52.36	405.36

Table S2. Most abundant proteins within matrisome (native kidney)

Uniprot Accession	Gene Name	Protein Name	Relative abundance (%)
A0A287ATP0	HSPG2	Heparan sulfate proteoglycan 2	16.74
A0A4X1TZA7	A2M	Alpha-2-Macroglobulin	9.17
A0A4X1VBD0	ANXA5	Annexin	6.36
A0A4X1UPI9	TINAG	SMB domain-containing protein	3.96
A0A4X1TYI6	LAMC1	Uncharacterized protein	3.95
F1RQI0	COL12A1	Collagen type XII alpha 1 chain	3.90
A0A4X1WE67	NID2	Nidogen-2	3.87
F1RMN7	HPX	Hemopexin	3.80
P19620	ANXA2	Annexin A2	3.71
A0A286ZY95	FN1	Fibronectin	3.50

Table S3. Most abundant proteins within matrisome (kidney dECM)

Uniprot Accession	Gene Name	Protein Name	Relative abundance (%)
A0A287ATP0	HSPG2	Heparan sulfate proteoglycan 2	30.03
A0A4X1TYI6	LAMC1	Laminin subunit gamma-1	10.37
A0A5G2RJ53	COL4A2	Collagen type IV alpha 2 chain	10.22
A0A4X1UPI9	TINAG	SMB domain-containing protein	7.62
A0A4X1SK88	FBN1	Fibrillin-1	6.47
F1SAE9	LAMB1	Laminin subunit beta 1	4.88
A0A4X1WE67	NID2	Nidogen-2	4.04
F1RQI0	COL12A1	Collagen type XII alpha 1 chain	3.99
I3LJT1	NID1	Nidogen 1	2.94
A0A4X1SG78	LUM	Lumican	1.98

Table S4. Real-time quantitative PCR primer list

Gene		Sequence (5'-3')
ABCB1	Forward	CCATGCTCAGACAGGATGTG
	Reverse	TTCCTGTCCCAAGATTTGCT
Angiotensin2 (ANG2)	Forward	ATA AGC AGC ATC AGC CAA CC
	Reverse	AAG TTG GAA GGA CCA CAT GC
ATP1A1	Forward	CCAATTGTGTTGAAGGCACC
	Reverse	CCGTGATGATGTGGATAAAATGT
COL1	Forward	CCC TGG AAA GAA TGG AGA TGA T
	Reverse	ACT GAA ACC TCT GTG TCC CTT CA
COL4	Forward	CGG GTA CCC AGG ACT CAT AG
	Reverse	GGA CCT GCT TCA CCC TTT TC
CRABP1	Forward	GCAGCAGCGAGAATTTCGAC
	Reverse	CGTGGTGGATGTCTTGATGTAGA
ECAD	Forward	TGCCCAGAAAATGAAAAAGG
	Reverse	GTGTATGTGGCAATGCGTTC
eNOS	Forward	CCT CGT CCC TGT GGA AAG AC
	Reverse	GAA TTG ACG CCT TCA CTC GC
Fibronectin	Forward	GGA GAA TTC AAG TGT GAC CCT CA
	Reverse	TGC CAC TGT TCT CCT ACG TGG
ITGA5	Forward	TGC AGT GTG AGG CTG TGT ACA
	Reverse	GTG GCC ACC TGA CGC TCT
ITGB1	Forward	GTG GCC ACC TGA CGC TCT
	Reverse	GTG GCC ACC TGA CGC TCT
ITGB4	Forward	GTG GCC ACC TGA CGC TCT
	Reverse	GGT CTC CTC TGT GAT TTG GAA
LRP2	Forward	TGTGATGCAGCCATCGAACT
	Reverse	TGCATTTGGGGAGGTCAGTC
MAP2	Forward	CTCAGCACCGCTAACAGAGG
	Reverse	CATTGGCGCTTCGGACAAG
MCAM	Forward	CGTCTCGTAAGAGCGAACTTG
	Reverse	CGATGTATTTCTCTCCCTGGTC
MYLPF	Forward	GAAGGACAGTAGAGGGCGGAA
	Reverse	TCTGGTCGATCACAGTGAAGG
NPHS1	Forward	GACCCAGCTTCCCATCACTA
	Reverse	GCATTGGAGAGGAGCAGAAG
PECAM1	Forward	TCATTACGGTCACAATGACGA
	Reverse	GAGTATCTGCTTTCCACGGC
PKD1	Forward	AACAAGTCTTTGGCCATCAC
	Reverse	TACTCGTTCAGCACGGTGAC

PKD2	Forward	TCTTGCCAATTTCAGCCTTT
	Reverse	GCACAACGATCACAACATCC
PKHD1	Forward	CCATTCTCTGCCAGGTTAGC
	Reverse	ACCCCTAATCAGCACAGTGG
PMEL	Forward	AGGTGCCTTTCTCCGTGAG
	Reverse	AGCTTCAGCCAGATAGCCACT
PODXL	Forward	GATAAGTGCGGCATACGGCT
	Reverse	GCTCGTACACATCCTTGGCA
SLC34A1	Forward	TCACGAAGCTCATCATCCAG
	Reverse	TTCCTCAGGGACTCATCACC
SOD2	Forward	TAG GGC TGA GGT TTG TCC AG
	Reverse	TAG GGC TGA GGT TTG TCC AG
WT1	Forward	GGGTACGAGAGCGATAACCA
	Reverse	TCTCACCAGTGTGCTTCCTG

Table S5. RNA-seq analysis of differentially expressed genes (10<DEGs) in kidney organoid cells

Cell type	Enriched genes	Marker
**SSB pro, S-shaped body proximal segment**	PCP4, CDH6, IFITM3, LAMP5, PAX8, LYPD1, KRT18, KRT8, IGFBP7, SST	IGFBP7, LAMP5
**Podocyte**	NPHS1, NPHS2, PODXL, MAF8, CTGF, PTPRO, GADD45A, BST2, S100A6, CPXM1, SOST	PODXL, NPHS1
**SSB pod, S-shaped body podocyte precursor**	PBK, HMMR, OLFM3, FOXC2, MKI67, NUSAP1, CENPF, TPX2, TOP2A, SMC4, HMGB2, PTTG1, TUBA1B, HIST1H4C	PBK, OLFM3, FOXC2, HMMR
**TP, Tubule progenitor**	SPP1, FTL, CAMK2N1, MPC2, CLU, ARL4C, ID1, PTGR1, APOE, CKB	SPP1, ID1
**PT, Proximal tubule**	HPN, SPP1, AFP, MT1F, TMEM176A, MT1E, MT1G, MT1X, MT2A, APOE, FTL, MT1H	HPN, SPP1
**LOH, Loop of Henle**	WFDC2, MAL, TMEM52B, MAL, DEFB1, LIMCH1, MECOM, HOXD8, GNG11, SAT1, ARL4C	WFDC2, MAL
**SSB md, S-shaped body medial and distal segnemt**	PAX2, H2AFZ, HMGA1, CENPF, TOP2A, TUBA1B, ARL4C, PTTG1, HMGB2, WFDC2, HIST1H4C	HMGA1, PAX2
**Mesen, Mesenchyme**	IGFBP5, IGFBP3, COL3A1, COL1A1, SFRP1, AKAP12, CRABP2, PBX1, IGF2, SST	COL1A1, COL3A1
**Endothelium1**	SOX18, ECSCR, IL32, TMSB4X, GNG11, TMSB10, CALM1, EGFL7, MYL12A, TPM3, FAM107B, FKBP1A	SOX18, ECSCR
**Endothelium2**	CD34, KDR, GJA4, ESM1, CD93, ESAM, RAMP2, EGFL7, TCF4, GNG11, CALM1, TMSB4X	CD34, KDR
**Glial**	RFX4, MSX1, SOX2, METRN, BEX1, TUBA1A, IGDCC3, LIMCH1, TTYH1, NEFM	MSX1, SOX2
**Melanoma**	PLP1, PMEL, TFAP2B, DCT, EDNRB, POSTN, LMO4, CRABP1, METRN, CENPF	PMEL, PLP1

**Neuron**	STMN2, TAGLN3, DLL3, CRABP1, HES6, TUBB2B, MAP1B, SOX4, CALM1, TUBA1A	DLL3, HES6
**RV.CSB**	FAM211A, CTNNB1, HNRNPH1, MT-ND1, EIF5A, ARL4C, HMGA1, MTRNR2L12, PTPRS	CTNNB1
**Precursor**	MTRNR2L12, 75K.2	MTRNR2L12
**Cell cycle**	NUSAP1, UBE2C, TOP2A, MKI67, HMGB2, H2AFZ, TUBA1B, HIST1H4C, HIST1H1B, HIST1H3D, PTTG1	TOP2A, MKI67, UBE2C,

Table S6. Summary of statistical analysis

	Comparison	N	P value	Significance
**Figure 2c**	Matrigel based protocol vs. Protocol A	10 (Organoid number)	<0.0001	Y
	Matrigel based protocol vs. Protocol B	10 (Organoid number)	<0.0001	Y
	Matrigel based protocol+VEGF vs. Protocol A	10 (Organoid number)	<0.0001	Y
	Matrigel based protocol+VEGF vs. Protocol B	10 (Organoid number)	<0.0001	Y
**Figure 2e**	Matrigel based protocol vs. Protocol A	3 (Experiment number)	0.0201	Y
	Matrigel based protocol vs. Protocol B	3 (Experiment number)	<0.0001	Y
	Matrigel based protocol+VEGF vs. Protocol A	3 (Experiment number)	0.0308	Y
	Matrigel based protocol+VEGF vs. Protocol B	3 (Experiment number)	<0.0001	Y
	Protocol A vs. Protocol B	3 (Experiment number)	0.0024	Y
**Figure 2g**	Matrigel based protocol vs. Protocol B	3 (Experiment number)	<0.0001	Y
	Matrigel based protocol+VEGF vs. Protocol B	3 (Experiment number)	<0.0001	Y
	Protocol A vs. Protocol B	3 (Experiment number)	<0.0001	Y
**Figure 2i**	Matrigel based protocol vs. Matrigel based protocol + VEGF	3 (Experiment number)	0.0089	Y
	Matrigel based protocol vs. Protocol A	3 (Experiment number)	<0.0001	Y
	Matrigel based protocol vs. Protocol B	3 (Experiment number)	<0.0001	Y
	Matrigel based protocol + VEGF vs. Protocol A	3 (Experiment number)	<0.0001	Y
	Matrigel based protocol + VEGF vs. Protocol B	3 (Experiment number)	<0.0001	Y
	Protocol A vs. Protocol B	3 (Experiment number)	<0.0001	Y
**Figure 2j**	Matrigel based protocol vs. Protocol A	3 (Experiment number)	<0.0001	Y
	Matrigel based protocol vs. Protocol B	3 (Experiment number)	<0.0001	Y
	Matrigel based protocol + VEGF vs. Protocol A	3 (Experiment number)	<0.0001	Y
	Matrigel based protocol + VEGF vs. Protocol B	3 (Experiment number)	<0.0001	Y
	Protocol A vs. Protocol B	3 (Experiment number)	<0.0001	Y
**Figure 3b**	Matrigel based protocol vs. Matrigel based protocol+VEGF	3 (Experiment number)	0.9633	N
	Matrigel based protocol vs. Protocol A	3 (Experiment number)	0.0003	Y
	Matrigel based protocol vs. Protocol B	3 (Experiment number)	<0.0001	Y
	Matrigel based protocol+VEGF vs. Protocol A	3 (Experiment number)	0.0005	Y
	Matrigel based protocol+VEGF vs. Protocol B	3 (Experiment number)	<0.0001	Y
	Protocol A vs. Protocol B	3 (Experiment number)	0.0436	Y
**Figure 4c**	Matrigel based protocol vs. Protocol A	4 (Experiment number)	0.0384	Y
	Matrigel based protocol vs. Protocol B	4 (Experiment number)	0.0004	Y
	Protocol A vs. Protocol B	4 (Experiment number)	0.0230	Y
**Figure 4e**	Matrigel based protocol vs. Protocol A	4 (Experiment number)	0.0029	Y
	Matrigel based protocol vs. Protocol B	4 (Experiment number)	<0.0001	Y
	Protocol A vs. Protocol B	4 (Experiment number)	0.0337	Y
**Figure 4f**	PKD1 : Matrigel based protocol vs. Protocol A	3 (Experiment number)	0.0008	Y
	PKD1 : Matrigel based protocol vs. Protocol B	3 (Experiment number)	<0.0001	Y
	PKD1 : Protocol A vs. Protocol B	3 (Experiment number)	0.5977	N
	PKD2 : Matrigel based protocol vs. Protocol A	3 (Experiment number)	0.1241	N
	PKD2 : Matrigel based protocol vs. Protocol B	3 (Experiment number)	0.0005	Y
	PKD2 : Protocol A vs. Protocol B	3 (Experiment number)	0.0409	Y
	PKDH1 : Matrigel based protocol vs. Protocol A	3 (Experiment number)	<0.0001	Y
	PKDH1 : Matrigel based protocol vs. Protocol B	3 (Experiment number)	<0.0001	Y
	PKDH1 : Protocol A vs. Protocol B	3 (Experiment number)	0.9935	N
	SLC34A1 : Matrigel based protocol vs. Protocol A	3 (Experiment number)	<0.0001	Y

Figure 4f	SLC34A1 : Matrigel based protocol vs. Protocol B	3 (Experiment number)	<0.0001	Y
	SLC34A1 : Protocol A vs. Protocol B	3 (Experiment number)	0.1511	N
	ATP1A1 : Matrigel based protocol vs. Protocol A	3 (Experiment number)	0.0002	Y
	ATP1A1 : Matrigel based protocol vs. Protocol B	3 (Experiment number)	0.0362	Y
	ATP1A1 : Protocol A vs. Protocol B	3 (Experiment number)	0.0218	Y
	ABCB1 : Matrigel based protocol vs. Protocol A	3 (Experiment number)	<0.0001	Y
	ABCB1 : Matrigel based protocol vs. Protocol B	3 (Experiment number)	0.2178	N
	ABCB1 : Protocol A vs. Protocol B	3 (Experiment number)	<0.0001	Y
	LRP2 : Matrigel based protocol vs. Protocol A	3 (Experiment number)	<0.0001	Y
	LRP2 : Matrigel based protocol vs. Protocol B	3 (Experiment number)	<0.0001	Y
	LRP2 : Protocol A vs. Protocol B	3 (Experiment number)	0.7892	N
	NPHS1 : Matrigel based protocol vs. Protocol A	3 (Experiment number)	<0.0001	Y
	NPHS1 : Matrigel based protocol vs. Protocol B	3 (Experiment number)	<0.0001	Y
	NPHS1 : Protocol A vs. Protocol B	3 (Experiment number)	<0.0001	Y
	WT1 : Matrigel based protocol vs. Protocol A	3 (Experiment number)	<0.0001	Y
	WT1 : Matrigel based protocol vs. Protocol B	3 (Experiment number)	0.0015	Y
	WT1 : Protocol A vs. Protocol B	3 (Experiment number)	0.4219	N
	PODXL : Matrigel based protocol vs. Protocol A	3 (Experiment number)	<0.0001	Y
	PODXL : Matrigel based protocol vs. Protocol B	3 (Experiment number)	<0.0001	Y
	PODXL : Protocol A vs. Protocol B	3 (Experiment number)	<0.0001	Y
Figure 4f	ECAD : Matrigel based protocol vs. Protocol A	3 (Experiment number)	<0.0001	Y
	ECAD : Matrigel based protocol vs. Protocol B	3 (Experiment number)	0.0115	Y
	ECAD : Protocol A vs. Protocol B	3 (Experiment number)	<0.0001	Y
Figure 4h	Matrigel based protocol vs. Matrigel based protocol+VEGF	3 (Experiment number)	0.9709	N
	Matrigel based protocol vs. Protocol A	3 (Experiment number)	0.0348	Y
	Matrigel based protocol vs. Protocol B	3 (Experiment number)	0.0002	Y
	Matrigel based protocol+VEGF vs. Protocol A	3 (Experiment number)	0.0640	N
	Matrigel based protocol+VEGF vs. Protocol B	3 (Experiment number)	0.0003	Y
	Protocol A vs. Protocol B	3 (Experiment number)	0.0094	Y
Figure 7e	Wild type vs. GLA mutant 2	3 (Experiment number)	0.0006	Y
	GLA mutant 2 vs. GLA mutant 2 + ERT	3 (Experiment number)	0.0045	Y
Figure 7h	SOD : Wild type vs. GLA mutant 2	3 (Experiment number)	0.03	Y
	eNOS : Wild type vs. GLA mutant 2	3 (Experiment number)	<0.0001	Y
	eNOS : Wild type vs. GLA mutant 2 + ERT	3 (Experiment number)	0.0002	Y
	ANG2 : Wild type vs. GLA mutant 2	3 (Experiment number)	0.0334	Y
Figure 8b	Transplanted organoids vs. Transplanted organoids with kideny dECM	6 (Organoid number)	0.0026	Y
Figure S2 b	Day 0 vs. Day 3	3 (Experiment number)	0.0210	Y
	Day 0 vs. Day 6	3 (Experiment number)	0.0017	Y
	Day 0 vs. Day 9	3 (Experiment number)	0.0005	Y
	Day 0 vs. Day 12	3 (Experiment number)	0.0003	Y
	Day 0 vs. Day 15	3 (Experiment number)	0.0001	Y
	Day 0 vs. Day 18	3 (Experiment number)	0.0001	Y
Figure S2 d	ITGB4 : Matrigel based protocol vs. Protocol A	3 (Experiment number)	<0.0001	Y
	ITGB4 : Matrigel based protocol vs. Protocol B	3 (Experiment number)	<0.0001	Y
	COL1 : Matrigel based protocol vs. Protocol B	3 (Experiment number)	<0.0001	Y
	COL1 : Protocol A vs. Protocol B	3 (Experiment number)	<0.0001	Y
	FN : Matrigel based protocol vs. Protocol B	3 (Experiment number)	<0.0001	Y
	FN : Protocol A vs. Protocol B	3 (Experiment number)	0.0177	Y
Figure S7 f	MAP2 : Matrigel based protocol vs Matrigel based protocol + Twist1 inhibitor	3 (Experiment number)	0.0003	Y
	CRABP1 : Matrigel based protocol vs Matrigel based protocol + Twist1 inhibitor	3 (Experiment number)	0.5552	N
	MYLPF : Matrigel based protocol vs Matrigel based protocol + Twist1 inhibitor	3 (Experiment number)	<0.0001	Y

**Figure S8 b**	Matrigel based protocol vs. Matrigel based protocol + Bevacizumab	3 (Experiment number)	0.9891	N
	Matrigel based protocol+VEGF vs. Matrigel based protocol+VEGF + Bevacizumab	3 (Experiment number)	0.2913	N
	Protocol A vs. Protocol A + Bevacizumab	3 (Experiment number)	0.0054	Y
	Protocol B vs. Protocol B + Bevacizumab	3 (Experiment number)	0.0005	Y

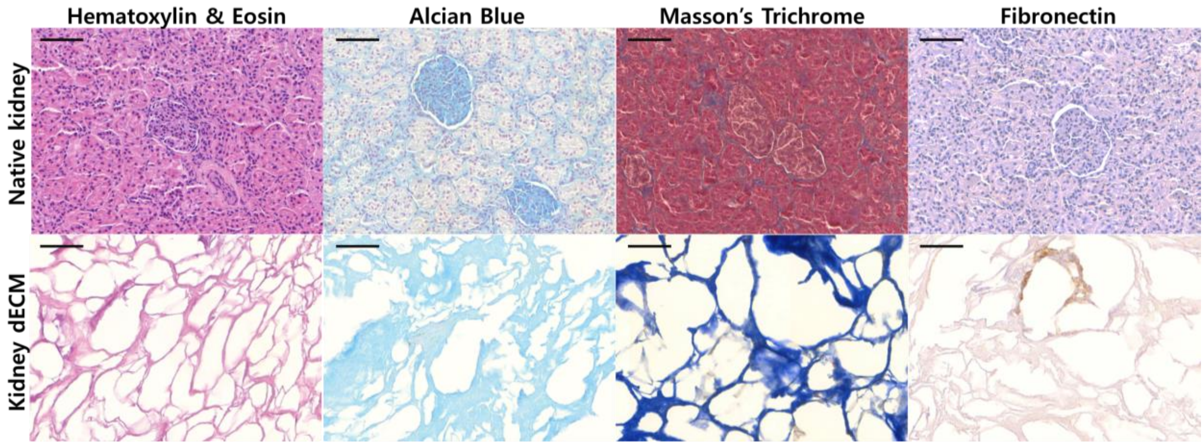

Figure S1. High-magnification (15x) images of kidney tissue before decellularization (Native kidney) and after decellularization (Kidney dECM) for H&E, Alcian Blue, Masson's Trichrome, and fibronectin. (Scale bar = 100μm)

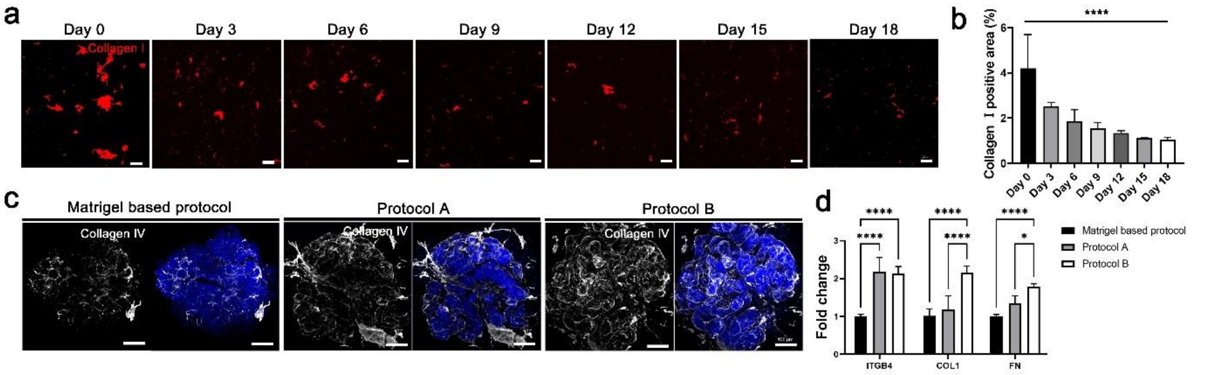

Figure S2. Monitoring the degradation nature of collagen I of the coated kidney dECM (a,b) and kidney dECM remodeling analysis (c,d) a) Representative fluorescence image of collagen I stained by date on a confocal plate coated with 0.1% kidney dECM. b) Quantification of the percentage of collagen I positive area. (n=3). Values are mean ± SD. ****, p<0.0001, measured by One-way ANOVA with Dunnett's multiple comparisons test. c) Representative fluorescence image of collagen IV of kidney organoids cultured by Matrigel-based protocol, protocol A and protocol B. d) qRT-PCR analysis of Integrin receptors (ITGB4), Fibronectin (FN), Collagen I. (n=3). Values are mean ± SEM. *, p<0.05, ****, p<0.0001, measured by two-way ANOVA with Tukey's multiple comparisons test.

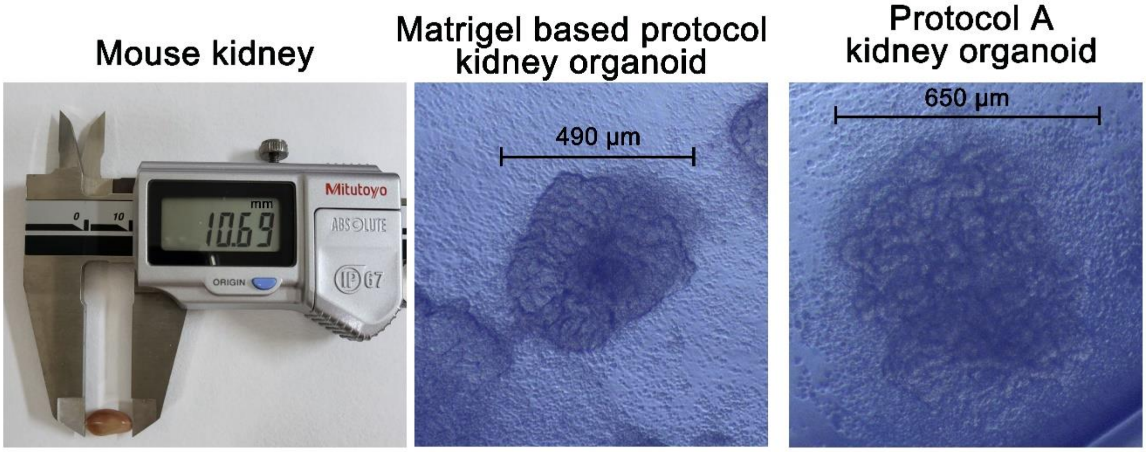

Figure S3. Comparison of mouse kidney and kidney organoid size. The mouse kidney size is 10.69 mm, the kidney organoid differentiated in Matrigel based protocol is 490 μm and the size of the kidney organoid differentiated in protocol A is 650 μm.

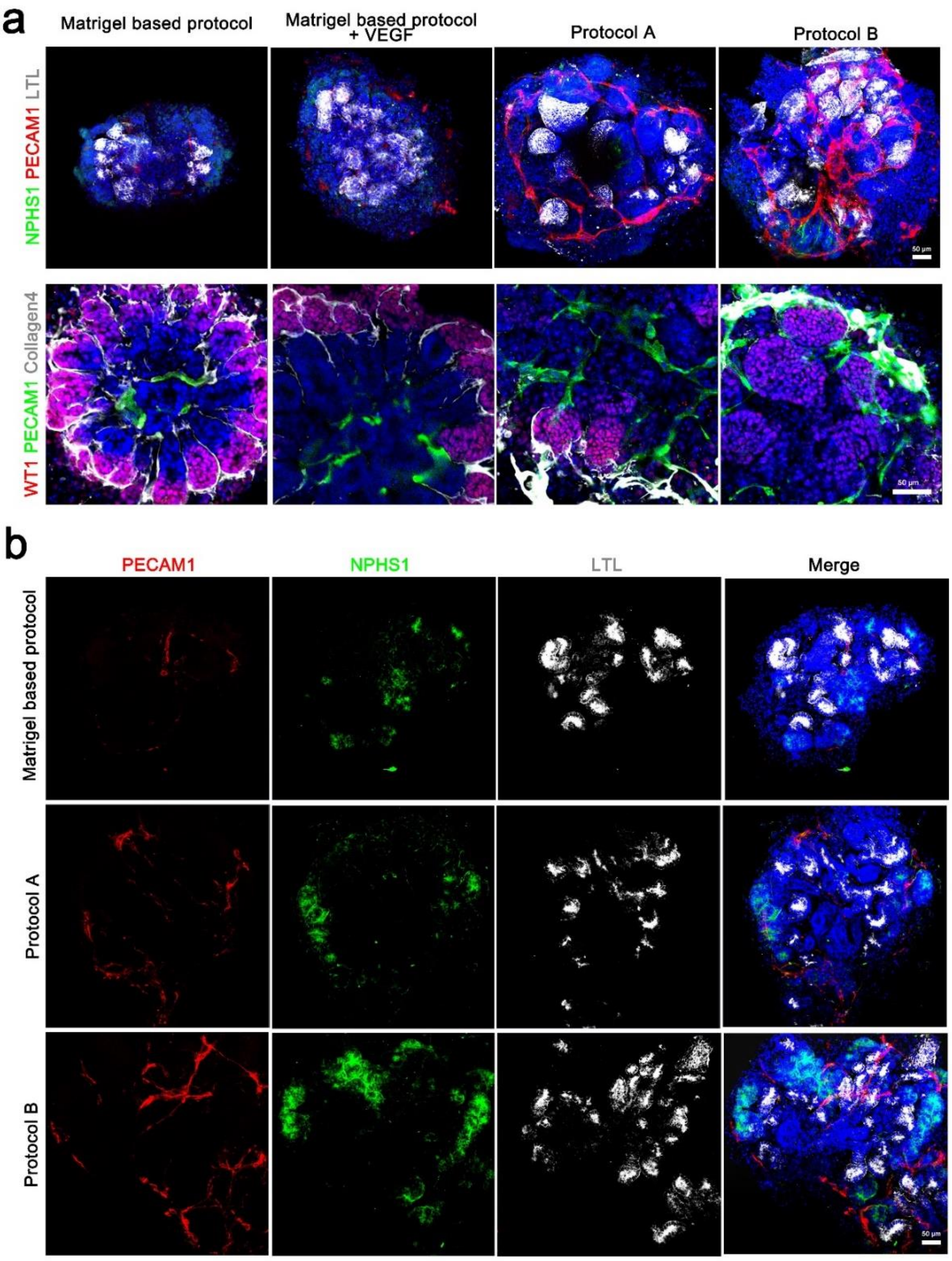

Figure S4. Low magnification images and images of immunofluorescent staining form CMC11 human pluripotent stem cell line. a) Representative low magnification images of immunofluorescent staining of NPHS1, PECAM1 and LTL in Figure 2d and WT1, PECAM1 and Collagen IV in Figure 2f. Scale bar = 50 µm. b) Kidney organoids differentiated from CMC11 iPSC cell line. Representative images of immunofluorescent staining of PECAM1, NPHS1 and LTL. Scale bar = 50 µm.

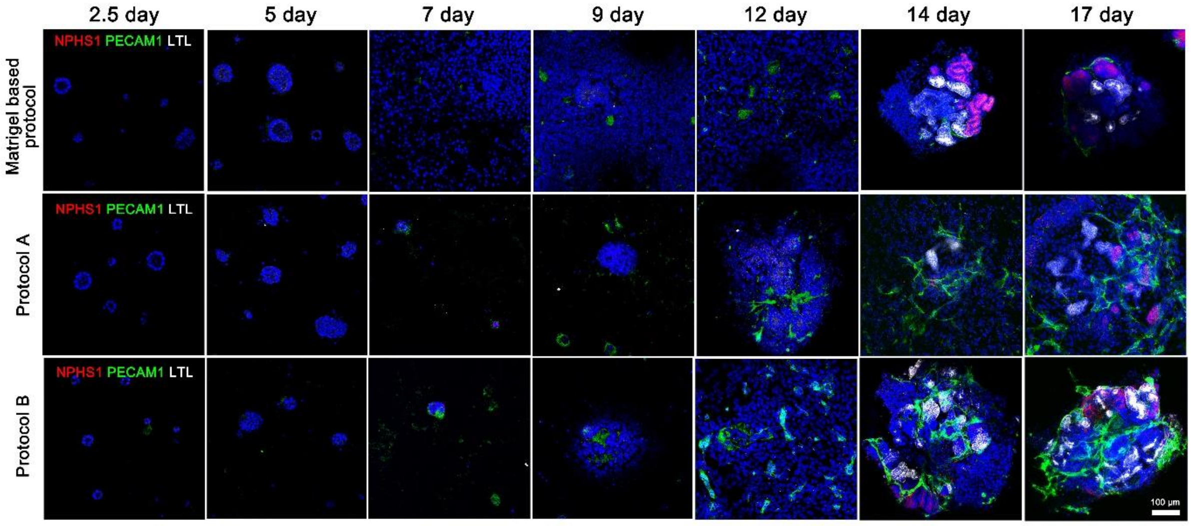

Figure S5. Monitoring the time of formation of endogenous PECAM1+ endothelial cells during the kidney organoids culture. Representative fluorescence image of NPHS1, PECAM1 and LTL from 2.5 to 17 days of kidney organoid differentiation.

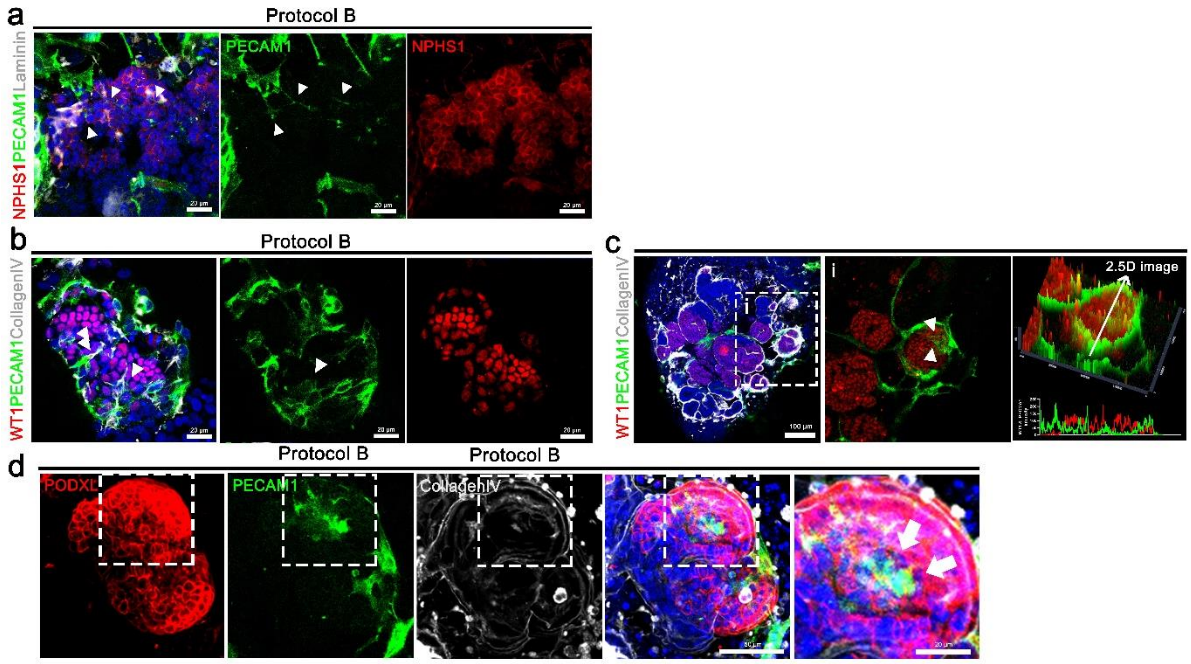

Figure S6. Enhanced vascularization of kidney organoids in protocol B. a) Representative images of immunofluorescent staining of NPHS1, PECAM1 and Laminin in kidney organoids. Invasion of PECAM1+ endothelial cells into NPHS1+ podocytes clusters (white arrowhead). Scale bar = 20 μm. b, c) Representative images of immunofluorescent staining of WT1, PECAM1 and Collagen IV in kidney organoids differentiated by protocol B. Invasion of PECAM1+ endothelial cells into WT1+ podocytes clusters (white arrowhead). Scale bar = 20 μm for b. Line scan of PECAM1 and WT1 showed line scan showing the invasion of PECAM1+ endothelial cells into WT1+ podocytes clusters in the panel (i) (white arrowhead). Scale bar = 100 μm for c. d) Representative images of immunofluorescent staining of PODXL (podocalyxin), PECAM1 and Collagen IV. White arrow indicated apico-basal polarity. Scale bar = 50 μm and 20 μm.

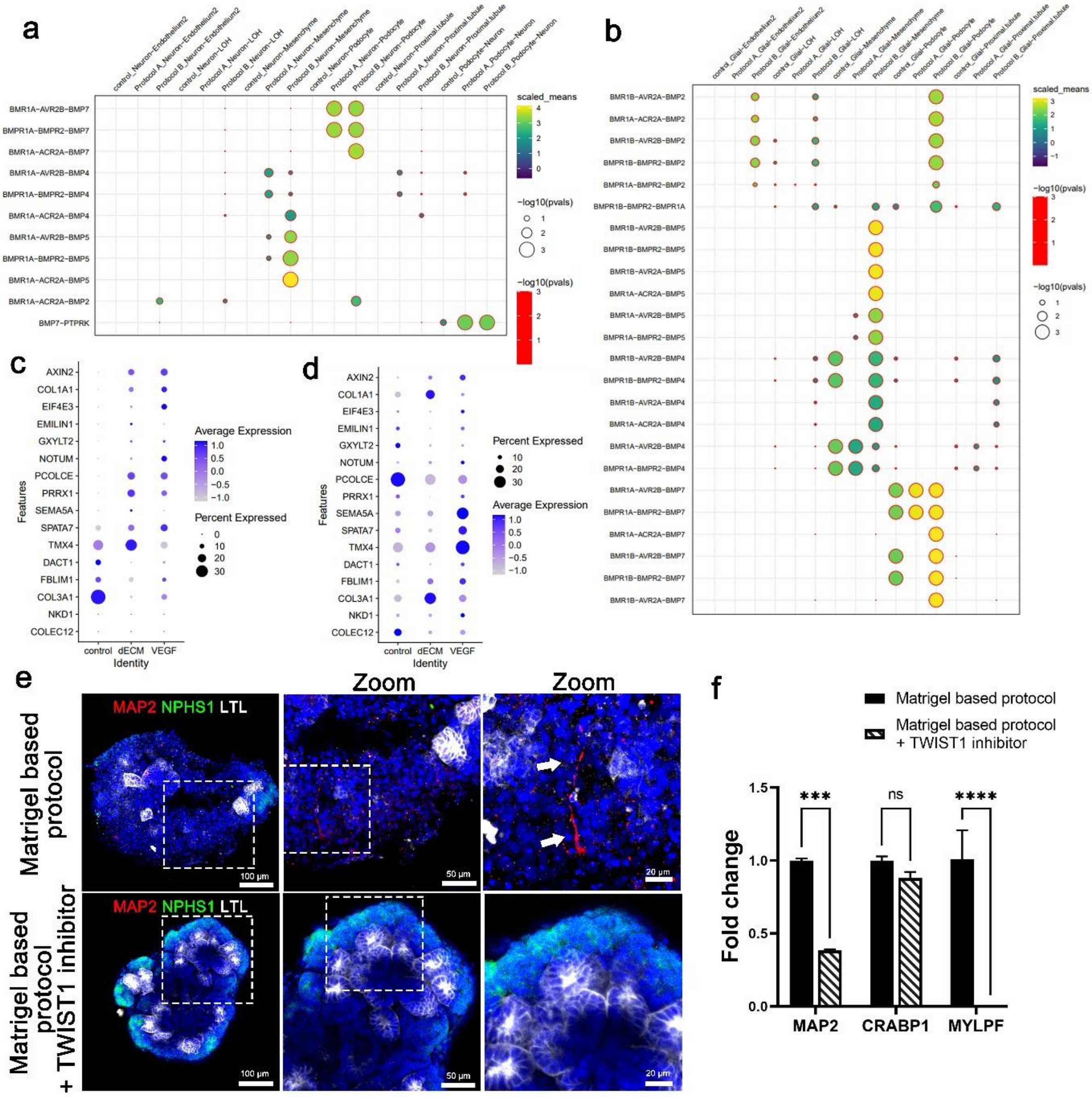

Figure S7. Analysis of the expression level of TWIST1 regulon in neurons and glial cells by RNA-seq analysis and treatment with TWIST1 inhibitor in kidney organoids. a) Bubble plot showing the BMP interaction in the neurons of kidney organoid in protocols A and B. b) Bubble plot showing the BMP interaction in the glial cells of kidney organoid in protocols A and B. c) Bubble plot showing the TWIST1 regulon expression levels in neurons. d) Bubble plot showing the TWIST1 regulon expression levels in glial cells. e) Immunofluorescent images of MAP2, NPHS1 and LTL staining after TWIST1 inhibitor (Harmine) treatment on kidney organoids differentiated in Matrigel-based protocol. f) qRT-PCR analysis of MAP2, CRABP1 and MYLPF. (n=2). Values are mean ± SEM. NS, no significance, ***, p<0.001, ****, p<0.0001, measured by Two-way ANOVA with Šídák's' multiple comparisons test

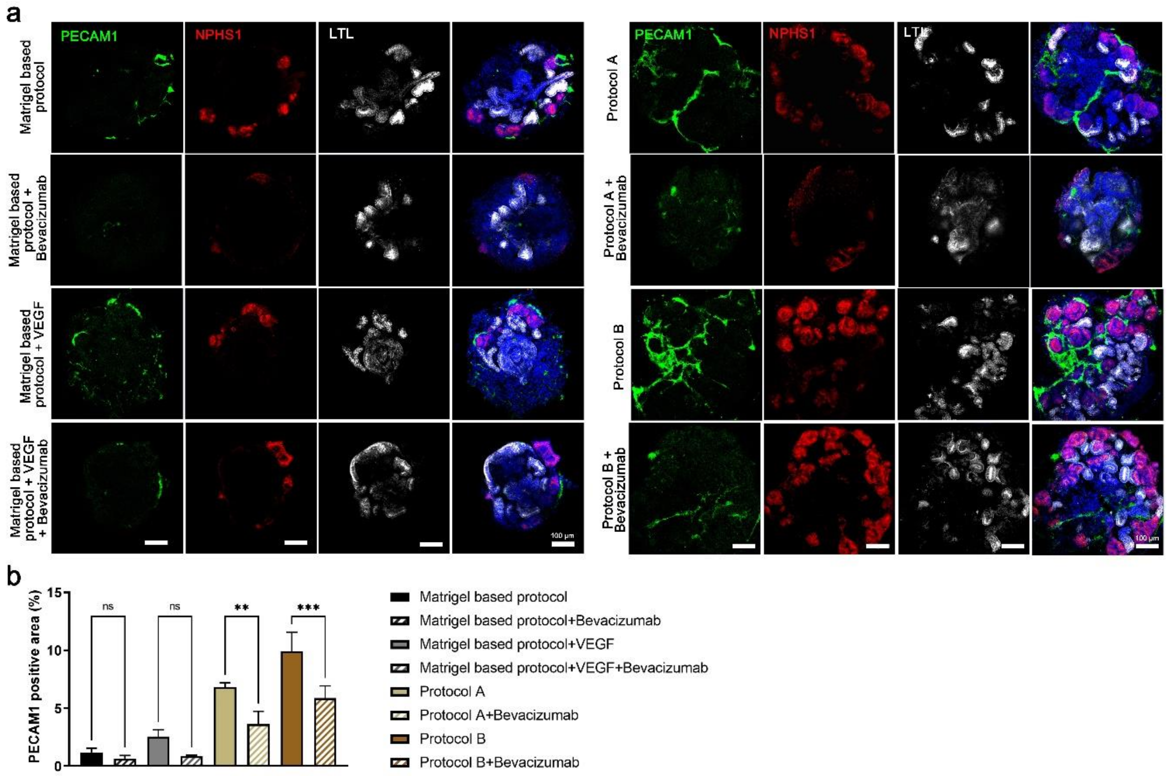

Figure S8. VEGF inhibitor-treated kidney organoids according to the differentiation protocols. a) Immunofluorescent images of PECAM1, NPHS1 and LTL staining after VEGF inhibitor (Bevacizumab) treatment on kidney organoids of four groups. b) Quantification of the percentage of PECAM1 positive area. (n=3). Values are mean ± SEM. **, $p<0.01$, ***, $p<0.001$, measured by t test.
